# RBC-GEM: A genome-scale metabolic model for systems biology of the human red blood cell

**DOI:** 10.1371/journal.pcbi.1012109

**Published:** 2025-03-12

**Authors:** Zachary B. Haiman, Alicia Key, Angelo D’Alessandro, Bernhard O. Palsson

**Affiliations:** 1 Department of Bioengineering, University of California San Diego, La Jolla, California, United States of America; 2 Department of Biochemistry and Molecular Genetics, University of Colorado Anschutz Medical Campus, Aurora, Colorado, USA; 3 Department of Pediatrics, University of California San Diego, La Jolla, California, United States of America; 4 Bioinformatics and Systems Biology Program, University of California, La Jolla, San Diego, California, United States of America; Clemson University, UNITED STATES OF AMERICA

## Abstract

Advancements with cost-effective, high-throughput omics technologies have had a transformative effect on both fundamental and translational research in the medical sciences. These advancements have facilitated a departure from the traditional view of human red blood cells (RBCs) as mere carriers of hemoglobin, devoid of significant biological complexity. Over the past decade, proteomic analyses have identified a growing number of different proteins present within RBCs, enabling systems biology analysis of their physiological functions. Here, we introduce RBC-GEM, one of the most comprehensive, curated genome-scale metabolic reconstructions of a specific human cell type to-date. It was developed through meta-analysis of proteomic data from 29 studies published over the past two decades resulting in an RBC proteome composed of more than 4,600 distinct proteins. Through workflow-guided manual curation, we have compiled the metabolic reactions carried out by this proteome to form a genome-scale metabolic model (GEM) of the RBC. RBC-GEM is hosted on a version-controlled GitHub repository, ensuring adherence to the standardized protocols for metabolic reconstruction quality control and data stewardship principles. RBC-GEM represents a metabolic network is a consisting of 820 genes encoding proteins acting on 1,685 unique metabolites through 2,723 biochemical reactions: a 740% size expansion over its predecessor. We demonstrated the utility of RBC-GEM by creating context-specific proteome-constrained models derived from proteomic data of stored RBCs for 616 blood donors, and classified reactions based on their simulated abundance dependence. This reconstruction as an up-to-date curated GEM can be used for contextualization of data and for the construction of a computational whole-cell models of the human RBC.

## Author summary

Human red blood cells (RBCs) have been studied for decades because of their unique physiology, essential oxygen delivery functions, and general accessibility. RBCs are the simplest of human cell types due to the loss of cellular organelles during their development process. This process has evolved to maximize hemoglobin content per cell to facilitate RBCs’ main function in gas transport. RBCs are integral to a variety of medical applications, such as blood storage for transfusion. Recent advancements in high-throughput data collection have greatly expanded our understanding of RBC metabolism, highlighting important roles and functions for RBCs in maintaining homeostasis in the organism in addition to oxygen transport. Here we provide a knowledge base for the human RBC as a genome-scale metabolic reconstruction. Our results highlight the complexity of RBC metabolism, supported by recent advancements in high-throughput data collection methods for detecting low-abundance proteins in RBCs. We make knowledge about the RBC findable, accessible, interoperable, and reusable (FAIR). We demonstrate how the reconstruction is converted into a mathematical model for modeling reaction rates based on protein abundances. We apply the model to contextualize proteomic data for over 600 blood donors whose RBCs were stored for 10, 23, and 42 days before sample acquisition. We subsequently classify reactions based on the strength of the correlation between maximum reaction rate within the network context and enzymatic abundance. As RBC research is likely to see many translational medical advancements, a platform for the contextualization of RBC data will serve as an essential resource for further research and medical application development.

## Introduction

Recent estimates suggest that red blood cells (RBCs) are by far the most numerous cell-type in the human body, accounting for ~83% of total human cells in an adult. The average lifespan of a healthy human RBC is approximately 120 days, during which the RBC undergoes approximately 200,000 circulatory cycles [1]. Each transit cycle takes approximately one minute. Thus, RBCs are subjected to constantly changing environmental stresses that exacerbate damage and degradation of proteins, from elevated oxidant stress in the lung to hypoxia and shear stress when traversing capillaries as narrow as 5 μm [2]. The RBC lacks the machinery necessary to synthesize new proteins de novo; consequently, the RBC proteome loses functionality over time, affecting essential functions in gas transport. Irreversible modifications to metabolic enzymes at key functional residues have been documented as a function of RBC aging *in vivo* and *in vitro*, ultimately promoting proteasome-dependent degradation of rate-limiting enzymes in key energy and redox metabolic pathways (e.g., glyceraldehyde 3-phosphate dehydrogenase – GAPDH or glucose 6-phosphate dehydrogenase – G6PD) [3–5]. Furthermore, both genetic and environmental factors influence irreversible glycation of hemoglobin. Consequently, the level of glycated hemoglobin has become a clinically important biomarker for glycemic control [6–8].

The relative simplicity of the RBC with respect to other cell-types, the lack of organelles, and its central, yet specialized role in systems physiology led to the consensus view that RBCs are inert cells with limited metabolic capabilities. RBC metabolism is tailored to sustain survival in circulation, deformability through the circulatory system, and, above all, the metabolic-dependent regulation of oxygen kinetics. As such, the focus on RBC metabolism has historically been limited to a subset of metabolic pathways, including energy metabolism via glycolysis, oxidative stress handling by the glutathione systems and the pentose phosphate pathway, and the allosteric regulation of hemoglobin oxygen-binding affinity through 2,3-bisphosphoglycerate (2,3BPG). However, two decades of proteomic studies have now elucidated an unexpected complexity of the RBC proteome, prompting a reevaluation of the purported simplicity of RBC metabolism. Despite a wealth of accumulating data, a systematic review of the literature and its organization according to latest standards in the field of systems biology is currently missing, creating the impetus for the current study.

Genome-scale reconstructions of metabolism are organized and systematized knowledge-bases of metabolism that serve as platforms to integrate multiple biological data types [9]. They can be converted into mathematical genome-scale metabolic models (GEMs) that are subsequently interrogated and interpreted within a metabolic context. Human GEMs contain all metabolic reactions known to occur across multiple human cell-types, encompassing all possible metabolic genes defined in the human genome without regard for tissue or cell specificity [10, 11]. Several cell-specific and/or context-specific human GEMs can be derived by mapping transcriptomic data or proteomic data onto these network reconstructions. GEMs derived from these data types are well-suited for the analysis of biological functions [12].

The first proteomically informed metabolic reconstruction of the human RBC, iAB-RBC-283 [13], was derived from the first global human reconstruction, Recon1 [14]. The iAB-RBC-283 knowledge base has been successfully utilized in numerous applications, including the development of personalized whole-cell kinetic models of RBCs [15], the elucidation of temperature dependence of RBC metabolism [16], and the exploration of host-parasite metabolic interactions in Plasmodium falciparum-infected RBCs [17, 18]. Several proteomics studies were utilized in the construction of iAB-RBC-283 with the largest study listing approximately 1,500 proteins identified in the erythrocyte [19–22]. Despite this expanded coverage, connectivity analysis of iAB-RBC-283 highlighted the need for targeted studies on the functionality of non-canonical citric acid cycles in the RBC, once believed to be inactive in enucleated RBCs [23]. Subsequent follow-up studies did indeed confirm the activity of several enzymes involved in citrate metabolism [24, 25].

With the increasing sensitivity and accuracy of proteomic approaches, the number of proteins identified in RBCs has now grown to over 3,000 [26, 27], prompting the need to generate a new reconstruction that encompasses such substantial progress. Much of the metabolic complexity associated with RBCs is within the low-abundance proteome. Advances in protein quantification strategies, driven by the increased sensitivity of latest generation mass spectrometers and the development of novel bioinformatics tools, have made it possible to estimate the copy numbers of the low-abundance proteins [28, 29]. The innovation and advances in affordable, high-throughput omics technologies continue to drive change in blood science and personalized transfusion medicine. These advancements promote the paradigm shift away from the long held views that RBCs are relatively inert cells [30–33], and “not a hapless sack of hemoglobin”, as Greenwalt put it [34]. There is a pressing need for an updated RBC knowledge base that reflects the last decade of advances made in RBC omics research. Furthermore, it is essential for improvements to be made in a tractable and transparent manner, as new technologies and methodologies expand our knowledge of the true scope of RBC metabolism with immediate, translational implications for human biology.

Here we present RBC-GEM, an updated knowledge base of erythrocyte metabolism provided as a GEM. The RBC-GEM reconstruction was developed by leveraging proteomic data from 29 publications and through manually curating decades of experimental literature primarily pertaining to human RBCs. We integrated the GEM with GitHub version-control software and the MEMOTE suite for standardized GEM testing [35], adhering to both findability, accessibility, interoperability, and reusability (FAIR) principles for scientific data management [35–37] and minimum information requested in the annotation of biochemical models (MIRIAM) guidelines for annotation and curation of quantitative models [38]. RBC-GEM is one of the most comprehensive, curated reconstructions of a specific human cell type to-date. We describe key considerations throughout the iterative reconstruction process, explore the topology of the resulting metabolic network, and verify both presence and activity of enzymes through the multitude of collected evidence. We quantitatively validate RBC-GEM through simulation. Our goal was to construct a curated GEM for human RBC metabolism that could serve as a foundational framework for RBC research across a multitude of disciplines.

## Results

### Generation of the RBC-GEM reconstruction

**Fig 1:**
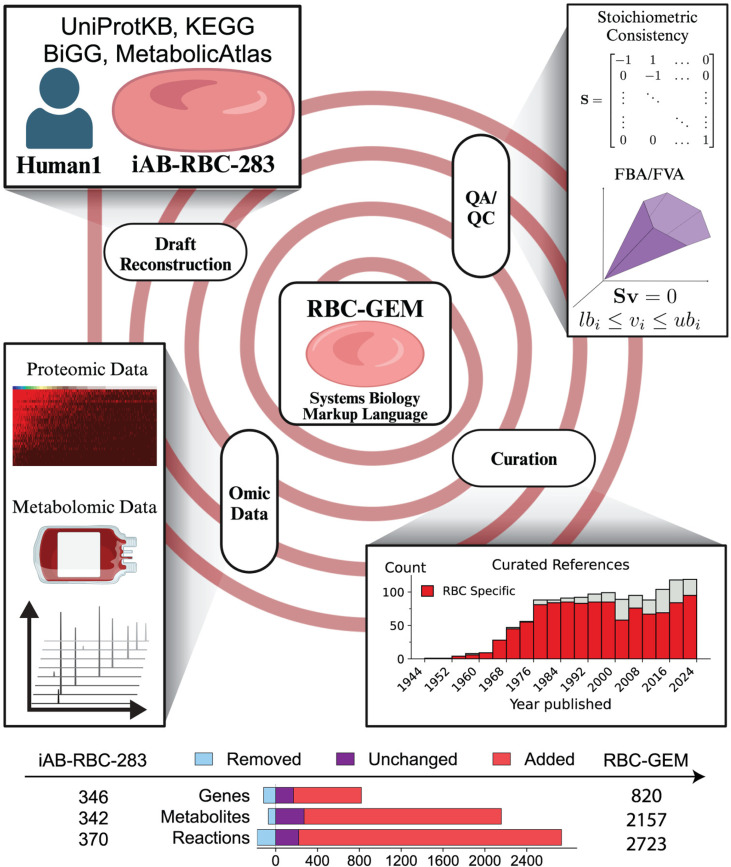
Workflow for the generation of RBC-GEM reconstruction. The RBC-GEM was generated through several cycles of iterative expansion and refinement. For the first cycle, iAB-RBC-283 [13] was downloaded from the BiGG Model database [41] as the initial reference network. New candidate reactions were identified by tailoring the global human reconstruction [39] for the RBC based on publicly available omic data and by cross-referencing the data with biochemical reaction and protein databases (e.g., KEGG [42], UniProtKB [43]). The existence and catalytic activity of enzymes were confirmed through systematic manual curation [9] of experimental literature with the vast majority directly pertaining to human RBCs. The final reconstruction is set up within a version-controlled and open-source framework for tractable and traceable improvements and was evaluated using the MEMOTE quality control (QC) utility [35]. Created with Biorender.com.

We began by reconciling the previously published iAB-RBC-283 reconstruction [13] with the current iteration of the Human-GEM reconstruction (version 1.19.0 [39]). We then followed the protocols developed for generating high-quality GEMs [9], while adhering to guidelines set for previously developed version-control frameworks for rapid, trackable model updates of consensus GEMs [35–37]. The final reconstruction, designated RBC-GEM, is made available in Systems Biology Markup Language (SBML) format (S1 File) and the BioModels database [40] (ID: MODEL2410170001). RBC-GEM was generated after subjecting the initial draft reconstruction to several refinement cycles guided by biochemical databases (Table A in S2 File), an abundance of publicly available proteomic data (Table B in S2 File and S1 Fig), rigorous manual curation (Table C in S2 File), and repetitive assessments of model quality (Fig 1). The RBC-GEM 1.2.0 reconstruction contained 820 genes (Table D in S2 File) encoding proteins acting on 2154 metabolites in which 1,685 are unique (Table E in S2 File). A total of 2,723 biochemical reactions are represented in the reconstruction: a 740% size expansion over its predecessor (Table F in S2 File).

### MIRIAM compliance

There is increasing consensus among the Systems Biology community that high-quality GEMs are constructed and distributed adhering to FAIR principles [35, 36]. The RBC-GEM was derived from both iAB-RBC-283 (downloaded from the BiGG Models database [41,44], and from Human-GEM (version 1.19.0 [39]), downloaded from the MetabolicAtlas [11]. Thus, for RBC-GEM to be findable, we utilized unique BiGG identifier standards and supplemented the reconstruction with existing MetabolicAtlas annotations where possible. New identifiers were created for reactions and metabolites that had ambiguous BiGG identifiers or were lacking them altogether. Gene identifiers were chosen based on their official symbols as defined by the HUGO Gene Nomenclature Committee (HGNC) database [45], and annotated with corresponding UniProtKB accessions. Unlike iAB-RBC-283, no isoform specificity was assigned to genes so that each gene uniquely represents one current and reviewed entry in the UniProtKB [43]. Through the UniProtKB ID mapping services, we were able to enrich the proteins represented in the reconstruction with compact identifiers for over 60 different databases.

We also enriched RBC-GEM with genetic and pharmacological information by mapping genes a total of 2712 drugs in DrugBank [46] (Table G in S2 File), 5055 single nucleotide polymorphisms (SNP) in UniProtKB [43] and SNP database [47] (Table H in S2 File) and 635 phenotypes of morbid SNPs from OMIM [48] (Table I in S2 File). Through the MetabolicAtlas annotations, we were also able to enrich metabolites and reactions with the annotations contained in Human-GEM. All annotations adhere to MIRIAM standards [38,49] that can be resolved through Identifiers.org [50]. However, several new additions for RBC-GEM, particularly those classified in the “Reactive species’‘ subsystem, are based primarily on RBC-specific literature [51, 52] and do not currently exist in Human-GEM. As our efforts for RBC-GEM 1.2.0 were focused primarily on expanding the proteomic coverage, these new metabolites and reactions were minimally annotated and present future areas of opportunity for the refinement of annotations.

### MEMOTE standardization

During the iterative reconstruction process (Fig 1), the reconstruction was periodically evaluated using the MEMOTE standardized testing suite that carries out quality control tests for metabolic reconstructions [35]. MEMOTE represents a community standard for assessing reconstruction through the application of a series of standardized set of tests and metrics. RBC-GEM demonstrated 100% stoichiometric consistency with all reactions mass-balanced, excluding pseudo-reactions such as boundary exchanges and the few reactions responsible for “pooling” individual lipid species into generic R-groups. Additionally, 99.8% of reactions are charge balanced with the only notable exceptions being the two reactions responsible for the reduction of methemoglobin, by either cytochrome b5 or flavin mononucleotide. The inability to balance these reactions is a likely consequence of simplifying the complexity of oxyhemoglobin binding and alpha-beta subunit interactions to a single subunit entity that could be treated as a metabolite within the reconstruction. We utilized flux balance/variability analysis (FBA/FVA) to ensure known and historically important metabolic pathways [53] remained functional. Dead-end metabolites and blocked reactions, especially those with literature evidence indicating functionality within RBCs *in vitro*, were left in the reconstruction because it is often unclear whether proteins are remnants of an imperfect degradation process or exhibit significant moonlighting functions [54]. The final reconstruction that was generated in this study is the largest in scope, omics and literature coverage and passes the major QA/QC tests with a MEMOTE score of approximately 83% (S3 File).

### RBC metabolism is contextualized and explored through network visualization

**Fig 2:**
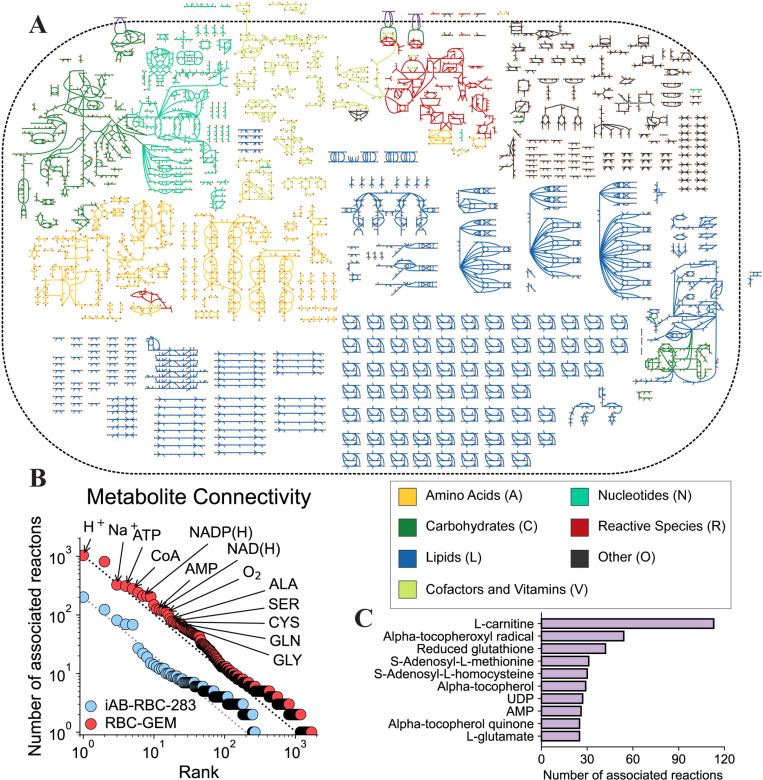
The expanded human RBC metabolic network. (A) The reconstructed RBC-GEM metabolic network is larger in scope and complexity than all previous erythrocyte models. (B) The resulting RBC metabolic network has a high connectivity with many points above the reference line, reflecting a more interconnected RBC metabolic network with over twice the proteomic coverage compared to its predecessor [13]. (C) Connectivity calculations were repeated after removal of highly connected cofactors, small ions, and transporters, elucidating subtle intrinsic trends in the intracellular network structure. Transport reactions were not included in the map for visual clarity. The network map was created using the Escher Network visualization tool [55].

In tandem with network reconstruction, we developed a network map of the full RBC metabolic network using the Escher Network visualization tool [55]. Visualization of biochemical pathways through detailed maps has enabled researchers to understand the conversion of metabolic species and coordination of enzymes within the cellular environment [56]. Through the Escher visualization tool, the entire RBC metabolic network can be visualized at once. We utilized KEGG pathways [42] to group the metabolic reactions into seven general categories (Table J in S2 File), which we subsequently visualized onto the network map (Fig 2A). We provide an interactive version of the map in which user-defined data can be overlaid onto it, providing visual context for the exploration of the RBC-GEM reconstruction and interpretation of data. Additionally, the map is provided as an Escher JSON file and in standard layouts generated by EscherConverter tool (S4 File). The accessibility of the map allows for the derivation of new user-generated pathway visualizations via Escher without having to start from a blank canvas. Furthermore, new maps can be deposited within the version-control framework of RBC-GEM where they can be maintained and reused across studies, aiding in the process of consensus building with respect to RBC metabolism.

### Metabolic pathways are connected through key cofactor pools

An open question from the reconstruction of iAB-RBC-283 was whether the low metabolite connectivity was a consequence of an inherently ‘fragmented’ erythrocyte network or incomplete proteomic coverage [13]. In RBC-GEM, we added hundreds of new genes, metabolites, and reactions to the iAB-RBC-283 reconstruction, supported by new multi-omic and experimental evidence. We compared the metabolite connectivity of RBC-GEM to its predecessor by connecting the minimum and maximum connectivity in each distribution with a reference line (Fig 2B). The distribution for iAB-RBC-283 was observed to reflect a less connected network, indicated by the number of points below the reference line [13]. Conversely, the distribution of connectivity for RBC-GEM reflected a network with higher connectivity as indicated by the number of points above the reference line. The increased coverage of the RBC metabolome in RBC-GEM is attributed to the utilization of multiple published omics datasets, many of which were published after iAB-RBC-283.

We computed the connectivity for each metabolite with and without regard for compartment boundaries (Table A in S5 File). The most connected metabolites were the cofactors that act as currency metabolites for energy metabolism (ATP, ADP, AMP) and redox metabolism (NADP(H), NAD(H)). RBCs rely on the glycolytic, pentose phosphate, and purine salvage pathways to maintain critical cofactor pools. Additional computational and metabolomic studies demonstrated that the citric acid cycle enzymes, such as isocitrate dehydrogenase (*IDH1*), malic enzyme (*ME1*), and malate dehydrogenase (*MDH1*), contributed to cofactor pools through regeneration of NAD(P)H and provision of intermediates to pyruvate kinase (*PKLR*) [24,25,32]. Conversely, numerous cellular processes depend on ATP hydrolysis for energy, while NAD(P)H is required for reduction of methemoglobin, the oxidized form of hemoglobin, through NADH-dependent cytochrome b5 reductase (*CYB5R3*) and NADPH-dependent flavin reductase (*BLVRB*), also known as biliverdin reductase B. As insufficient levels of ATP and NAD(P)H increase hemolytic propensity, understanding their connections to other metabolic pathways and the cause for their depletion is paramount for a multitude of biomedical research applications [32,51,53,57–60].

The expansion of lipid metabolism can be partly attributed to numerous, sparsely connected lipid species undergoing a similar set of reactions, transferring lipids between the highly connected Coenzyme A (CoA) and L-carnitine cofactors. The free and esterified lipid species form distinct, yet equilibrating pools that fuel and buffer phospholipid acyl-chain turnover as part of the Lands cycle [61–67]. The sodium ion (Na^+^) and amino acids have similar metabolite connectivity in both the intracellular and extracellular compartments, highlighting their involvement in membrane transport processes. Calculation of the gene connectivity (Table B in S5 File). confirmed the roles of the LAT1 heterodimer (*SLC7A5* and *SLC3A2*), a remnant of erythropoiesis with SNPs significantly associated with RBC kynurenine levels [68], and y+LAT2 heterodimer (*SLC7A6* and *SLC3A2*), initially identified via RBCs of individuals with Lysinuric Protein Intolerance [69, 70], in driving the exchange of amino acids across the membrane. As seen in other mammalian cell types, these transport proteins facilitate uptake of cationic and neutral amino acids into RBCs, which can be subsequently exported as a driving force for the Na^+^-dependent and Na^+^-independent uptake of other neutral amino acids [71].

Highly connected currency metabolites such as ATP and NADP(H) can dominant the network structure, masking intrinsic trends that may be more subtle [72]. Consequently, we modified reactions through removal of small ions and common substrate-product motifs, such as ATP hydrolysis and reduction via NADPH (Table C in S5 File). We performed additional connectivity calculations with the inclusion of transport reactions (Table D in S5 File) and with their removal (Table E in S5 File). Calculations with transport reactions demonstrated that most of the highly connected hubs were amino acids, agreeing with previous observations. Calculations after removal of transport reactions highlight import hubs in the smaller-scale intracellular network (Fig 2C). The most connected hubs include vitamin E due to its role as a lipid peroxyl radical scavenger [73], glutathione due to its functional role in conjugation reactions [74, 75], and the substrate-product pair of S-Adenosyl-L-methionine and S-Adenosyl-L-homocysteine for their involvement in methylation [4]. In both calculations with and without transport reactions, L-carnitine was the most connected metabolite. RBCs must maintain high deformability to squeeze through capillaries. However, decreased RBC deformability is a consequence of membrane damage induced by increased shear and oxidative stress, as observed in acute exercise [76]. Thus, acyl carnitines serve as a reservoir of activated acyl groups [61], becoming L-carnitine molecules as acyl carnitines are utilized in an ATP-independent manner for membrane remodeling via the Lands cycle [77, 78].

### Twenty years of proteomic data generation help define the reconstruction

**Fig 3:**
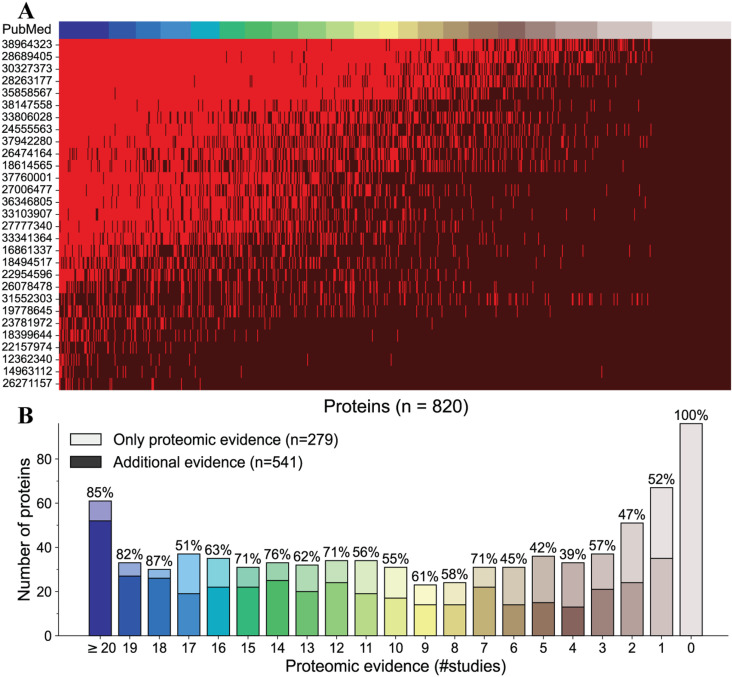
Proteomic and literature evidence for the RBC-GEM proteome. Proteomic data was collected across 29 datasets to facilitate the development of RBC-GEM. (A) The proteomic evidence for proteins defined in the RBC-GEM reconstruction is visualized as a binary heatmap representing the detection (bright red) or absence (dark red) of proteins across individual studies. (B). Manual curation provided additional evidence for 541 metabolic proteins in the reconstruction, with at least 80 proteins not detected in the proteomic evidence.

Transcriptomic and proteomic data is often utilized for the development of context-specific metabolic reconstructions for various cell types [12,79]. However, as human RBCs are devoid of nuclei, proteomic evidence was essential for developing the metabolic reconstruction, providing direct evidence for the existence of proteins. We collected published proteomic data for erythrocytes from 29 datasets [19–21,27–29,39,54, 80–100] that span 20 years of RBC proteomic research (Table B in S2 File and S1 Fig). Proteins that are detected consistently across the 29 proteomic studies were deemed as more likely to be part of the RBC proteome. Furthermore, proteomic technologies have only become more reliable over time [30] by coupling new technologies with emerging methodologies [84] for protein identification [80–83], quantification [28], and purity of mature RBC samples [29]. For this reason, we operated under the assumption that recently published proteomics data are more indicative of the mature erythrocyte proteome, and prioritized for mapping the collection of recent data sets designed for deep proteome profiling of the RBC cytosol and plasma membrane [27,28,87,88,91,92]. This wealth of proteomic data was mapped onto RBC-GEM 1.2.0 to gauge the level of supporting proteomic evidence for each protein in the updated reconstruction (Fig 3).

Proteomics data from highly purified reticulocyte populations and erythroid precursors were utilized to distinguish proteins that could be from a newly matured red blood cell from those that are clearly specific to immature erythroid precursors [29,91,101]. We also included a high-confidence canonical erythrocyte proteome defined by reconciling proteomic data through supervised machine learning [54]. There is increasing interest in using RBCs within the context of clinical proteomics [102] and transfusion medicine [30]; therefore, we included several studies that explore alterations in the RBC proteome due to various pathological disease states [85,90,93,95–97] and refrigerated storage under blood bank conditions [27,94,95], increasing the utility of RBC-GEM for biomarker discovery [103]. Because several proteomic datasets contained outdated and alternate accessions [19,20,80–84,86], the unavoidable consequence of routine database updates and previous database deprecations [104], we consolidated all identifiers into a list and utilized UniProtKB mapping service to update all protein identifiers to current UniProtKB accessions, pruning obsolete and unreviewed proteins from the list. In total, we found that over 4,600 proteins were detected at least once across all collected datasets (Table B in S2 File and S1 Fig).

### Metabolomics and literature curation verify enzymatic activity in the low-abundance proteome

While proteins that are detected consistently across studies are deemed to be part of the RBC proteome, it is important to note that the proteome may also include infrequently detected proteins. Differences in MS-instrumentation, fractionation strategies, contamination, and other sources of technical variability can lead to discrepancies in identified proteins. Functional metabolic tracing experiments suggest that there are still proteins in the RBC proteome that have yet to be identified [22,26,27,88]. Additionally, the presence of an enzyme does not necessarily indicate residual activity for that given enzyme. Therefore, both untargeted and tracing metabolomic experiments of human RBCs [4,24,27,105–110] were utilized to further substantiate the presence of enzymatic activity and validate the existence of related metabolites (substrates/products). By using high-throughput multi-omic data, we were able to gain a comprehensive understanding of the RBC proteome [30,31,111].

There are potential pitfalls that warrant caution when aggregating publicly available proteomic data for the identification of proteins in the RBC proteome. Over 80 proteins were found to have experimental evidence supporting their presence and activity in RBCs, yet they were not detected across all proteomic studies. For example, at least six different phosphodiesterase proteins have been identified in RBCs with important roles in regulating cyclic nucleotide levels [112], yet none were found in the proteomic data. Proteins involved in the synthesis of blood group antigens also do not appear across proteomic studies [113,114]. Even the most abundant proteins known to exist in the erythrocyte proteome (e.g., hemoglobin and the Band 3 anion exchanger) were not reported across all studies, perhaps because of high abundance protein depletion strategies aimed at unmasking the low-abundance proteome. The absence of evidence for a known RBC protein in a dataset may be caused by technical and methodological reasons, emphasizing the importance of detailed manual curation in the generation of RBC-GEM [4,9,24,27,105–107].

### Properties of the network reconstruction

**Fig 4:**
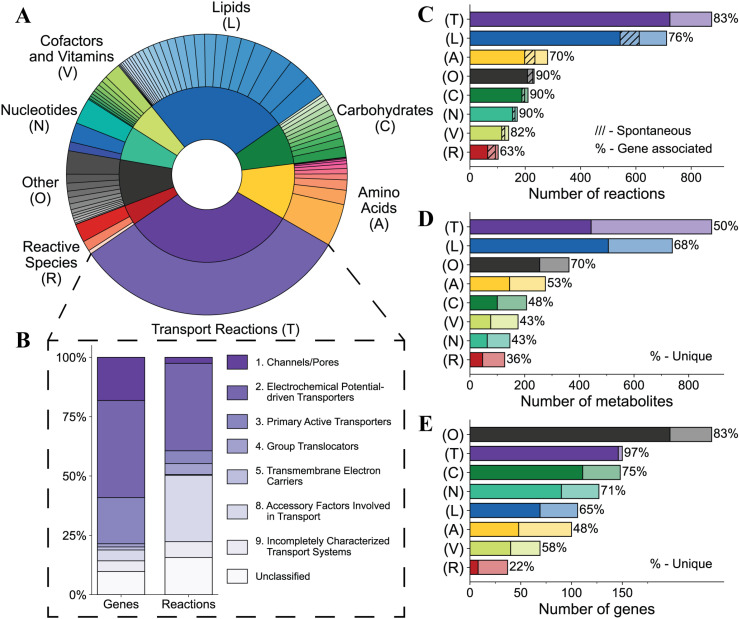
Assessment of RBC-GEM 1.2.0 properties through functional categorization of metabolic subsystems. The various metabolic subsystems represented in RBC-GEM were grouped into eight distinct categories. (A) The relative distribution of 76 metabolic subsystems across the generalized categories. Slice sizes in the inner and outer rings correspond to the number of reactions per category or per subsystem, respectively. (B) Transport reactions across the plasma membrane are further classified according to the Transport Classification System [115]. The “Unclassified” label is assigned to reactions associated with unknown or unclassified transport proteins as well as reactions representing passive diffusion. (C) The number of reactions per category. Darker colored regions represent reactions with gene associations, and hashed lighter regions represent known spontaneous reactions without gene associations. (D) The number of metabolites per category. Darker colored regions represent the percentage of metabolites unique to each category. (E) The number of genes per category. Darker colored regions represent the percentage of genes unique to each category. Metabolites and genes are defined as unique to a category if they are exclusively associated with reactions of the same category.

The properties of the RBC metabolic network are further contextualized through the functional categorization of reactions by their assigned subsystems (Fig 4). A subsystem is defined as a collection of functional roles that implement a specific biological process or structural complex. The set of functional roles that tie protein-encoding genes to different subsystems are known as subsystem connections [116]. RBC-GEM contains over 70 subsystems that can be classified into seven distinct metabolic categories with an additional category representing miscellaneous processes with varying physiological importance (Fig 4A). Because transport reactions in RBC-GEM are exclusive to one subsystem, we used the Transport Classification System [115] to classify membrane transporters (Fig 4B). Most reactions found within the RBC-GEM have known gene associations with a much smaller subset of reactions known to be spontaneous (Fig 4C). Most genes in RBC-GEM had one subsystem connection (Fig 4E), as is expected of protein-encoding genes [116]. However, genes found in the “Reactive Species” group were distributed across categories with superoxide dismutase (*SOD1*), methanethiol oxidase (*SELENBP1*), and sulfiredoxin (*SRXN1*), as notable exceptions. Furthermore, the representative metabolites were found across all aspects of cellular metabolism, hence the “Reactive Species” category contained the largest number of shared metabolites (Fig 4D), and genes (Fig 4E). These observations emphasize the need for both reactive species detoxification mechanisms across metabolic subsystems as well as the specific mechanisms for oxidative stress defense and repair [51,117,118].

### Hemoglobin allostery and pH modulate canonical metabolism

RBCs are well-known for their near exclusive reliance on glycolysis for the generation of ATP, the pentose phosphate pathway for NADPH regeneration, and the purine salvage pathway for maintaining adequate nucleotide concentrations. Many of these rate-limiting enzymes are pH sensitive, including hexokinase (*HK1*), phosphofructokinase isozymes (*PFKL*, *PFKM*, *PFKP*) [100], and *G6PD*. The product of glycolysis in RBCs is lactate [32], which exists in equilibrium with lactic acid at physiological pH. The increased lactate contributes to the Bohr effect via intracellular acidification, further promoting the oxygen off-loading from hemoglobin. Furthermore, *CYB5R3* competes with lactate dehydrogenase (LDH) for NADH, a phenomenon contributing to the accumulation of lactate observed in prolonged storage of RBCs [105,119].

The activity of the Rapoport-Luebering (RL) shunt is also pH sensitive and known to dictate the concentration of 2,3BPG in RBCs. At acidic pH, bisphosphoglycerate mutase (*BPGM*) phosphatase activity is dominant over synthase activity; conversely, at alkaline pH, the synthase activity is preferred [120]. RBCs also have multiple inositol polyphosphate phosphatase (*MIPP1*) with 2-phosphatase activity for 2,3BPG [121] and sensitivity to physiologic alkalosis, thus expanding the regulatory capacity of the RL shunt. The changes in 2,3BPG modulate hemoglobin allostery. The stabilization of deoxyhemoglobin in turn modulates the subcellular compartmentalization of glycolytic enzymes (phosphofructokinase – PFK; aldolase – *ALDOA*; *GAPDH*), which are bound to and inhibited by the N-terminus cytosolic domain of band 3 at high oxygen saturation. At low oxygen saturation, the bound glycolytic enzymes are outcompeted by deoxyhemoglobin and released. The release of glycolytic enzymes to the cytosol corresponds to an increase in the activity of these rate-limiting enzymes in the glycolytic pathway [100,122,123], forming an intricate feedback loop in which oxygen transport and delivery is finely regulated through RBC metabolic demands [124].

### Pathological metabolic states elucidate metabolic pathways

RBCs have several proteins, many exclusive to carbohydrate metabolism (Fig 4D) with metabolic functions for maintaining intracellular energy levels. Though RBCs can transport many carbohydrates across their membrane [125,126], they best utilize glucose to fuel energy metabolism with approximately 90% of glucose directed to glycolysis and the remaining 10% directed to the pentose phosphate pathway under normal, non-stress conditions [32,127]. Previous metabolomic experiments have revealed the ability of RBCs to metabolize citrate and other tricarboxylic acids [128] in addition to carbohydrates, such as fructose, mannose [106], and galactose [129]. Though not immediately visible in healthy individuals, RBCs from individuals with different forms of glycogen storage disease have revealed residual activity of synthesis and degradation enzymes [130]. Proteomic evidence also elucidated multiple metabolite repair enzymes in RBCs that have been shown to address side activities of glycolytic enzymes [131]. Use of this evidence in RBC-GEM is illustrated by the formation of methylglyoxal through oxidation of dihydroxyacetone phosphate (DHAP) [132,133], subsequent detoxification through the glyoxalase enzymes lactoylglutathione lyase (*GLO1*) and hydroxyacylglutathione hydrolase (*HAGH*), respectively known as Glyoxalase I and II [134], and deglycase activity of Parkinson disease protein 7 (*PARK7*) [135].

RBC nucleotide metabolism has been shown to be more complex than previously thought, as elucidated through pathological RBC states which are characterized by alterations in nucleotide patterns [136,137]. Nucleotide metabolism was composed of a few metabolic subsystems (Fig 4A) with nucleotide species found across the RBC metabolite network (Fig 4E) and regulated by specific enzymes to maintain the nucleotide pools (Fig 4D). RBCs have enzymes to salvage extracellular orotic acid to form uridine monophosphate (UMP) [138], to phosphorylate and dephosphorylate of deoxynucleotides [139,140], and salvage both nucleobase and ribose moieties of deoxynucleotides [141]. Additionally, erythrocytes have enzymes for uptake of cyclic nucleotides [142,143], signaling [112], degradation [144], and export [142]. Though early studies were unable to detect adenylosuccinate synthetase activity in RBCs [145], deep proteomic and metabolic tracing studies have demonstrated its presence and residual activity [146]. Furthermore, erythrocytes have the capacity to form unusual nucleotides, including the oncometabolite 4-pyridone-3-carboxamide-1-beta-D-ribonucleotides [147,148] and the Lesch Nyhan (LN) biomarker 5-Aminoimidazole-4-carboxamide ribonucleotides (AICAR) [97,147,148]. LN syndrome is an X-linked recessive inborn error caused by a pathogenic mutation in Hypoxanthine-Guanine Phosphoribosyltransferase (*HPRT1*). *HPRT1* is responsible for salvaging hypoxanthine, a catabolic product of ATP breakdown and deamination [97] by AMP deaminase 3 (*AMPD3*). LN is characterized by urate accumulation due to oxidation of excess hypoxanthine with concomitant generation of reactive oxygen species (ROS). Furthermore, oxidative stress is known to promote *AMPD3* activity while 2,3BPG inhibits it. Thus, the modulation of purine metabolism is intertwined with the RBC response to hypoxia via deregulation of *AMPD3* [146,149].

Erythrocytes are constantly exposed to ROS from external and internal sources of oxidants [51], and hemoglobin itself has been shown to participate in a variety of reactions outside of the standard roles it has in gas transport [52,150–153]. Therefore, we formed the “Reactive Species’‘ category with associated subsystems to represent hemoglobin-catalyzed reactions and auto-oxidation events that were likely to occur. Hemoglobin has displayed varying roles in systemic nitric oxide, sulfide, and redox metabolism [51,154]. The degradation of hemoglobin is non-enzymatic, generating reactive species [155] before glutathione facilitates degradation of hemin, as evident in hemolytic disorders like beta thalassemia [156,157]. Because hemoglobin is the most abundant protein in RBCs, we assumed that reactions shown to be catalyzed by hemoglobin *in vitro* were also possible *in vivo* and in blood storage conditions [52,150–153,158]. In addition to interactions with hemoglobin, we also included various reactive species detoxification reactions facilitated by intertwined antioxidant networks for “redoxins”, peroxiredoxins [159], thioredoxins [160], and glutaredoxins [161], in which they ultimately derive reducing power from NADPH [51]. The inclusion of several reactions involving the formation and detoxification of reactive oxygen, nitrogen, and sulfur species, especially in the context of their interactions with hemoglobin and “redoxins’‘ led to the formation of the “Reactive Species’‘ category [154].

In healthy human RBCs, the degradation of hemoglobin is a hallmark of aging RBCs. Increased ROS leads to a decreased capacity to reduce methemoglobin, resulting in increased damage to RBC membranes and release of glycerophosphocholine lipid products. Changes in availability of amino acids glycine, glutamine, and cysteine result in enzymatic activity diverted away from glutathione synthesis in favor of increased formation of the opthamalate side product, from glycine, glutamate, and the product of cysteine transamination, 2-aminobutyrate, [162]. Consequently, both glycerophosphocholine and opthamalate have been proposed candidate biomarkers for metabolic clocks in RBCs [162]. Thus, metabolic reactions associated with both metabolites were included in the RBC-GEM reconstruction to enable further exploration of the metabolic behavior.

### Lipid metabolism in RBCs is complex

Most spontaneous reactions in the lipid metabolism (Fig 4C) were due to the inclusion of lipid peroxidation reactions, in which hydroxyl species generated by Fenton and Haber-Weiss reactions initiate lipid peroxidation through hydrogen abstraction of representative polyunsaturated lipid species linoleate, arachidonate, eicosapentaenoate, and docosahexaenoate (C18:2, C20:4, C20:5, C22:6), followed by formation of the lipid peroxyl radical and eventual termination by vitamin E [51,73,157]. The generation of reactive species through oxidation of catechol estrogens and redox cycling due to *CYB5R3* was also included as estrogen may have profound roles in sex-based differences in blood storage [163–166]. Other updates to lipid metabolism in RBC-GEM reflect the erythrocyte incapacity for de novo synthesis of long chain fatty acids due to an inability to maintain a sufficient pool of Malonyl-CoA [167], instead relying on the Lands cycle for phospholipid remodeling and repair [77, 78].

Erythrocytes have demonstrated the ability to successively methylate phosphatidylethanolamine (PE) to phosphatidylcholine (PC) via an N-methyltransferase [168] and incorporate glucose, phosphate, glycerol, serine, and choline into phospholipids. RBCs also exhibited very low phosphatidylserine decarboxylase activity [169]. PC may serve as an unappreciated pool of methyl group donors to fuel protein-L-isoaspartate (D-aspartate) O-methyltransferase (*PCMT1*) and other methyltransferases for deamidation/dehydration-induced isoaspartyl-damage repair, as elucidated through metabolomics of stored RBCs [4,58,107]. Additionally, the abnormal phospholipid phosphatidylethanol was found almost exclusively in RBCs and may serve as a possible biomarker for alcohol consumption [170,171].

Sphingosine-1-phosphate (S1P), the bioactive signaling lipid that modulates the hypoxic response of RBCs [110,172], may experience limited degradation through low-activity of S1P degrading enzymes [173,174], but is primarily exported by the Sphingosine-1-phosphate transporter (*MFSD2B*) [175]. RBCs are capable of efficient sphingosine uptake and phosphorylation to SP via sphingosine kinase 1 (*SPHK1*) [176], enabling circulating RBCs to effectively serve as reservoirs for plasma S1P. RBCs have also demonstrated alkaline ceramidase, neutral and acid sphingomyelinase, and sphingomyelin synthase activities, indicating that sphingolipid metabolism in the membrane may represent another regulatory point for S1P metabolism [174]. RBC-GEM was updated to include the various metabolic processes for sphingolipid metabolism, thereby representing the processes for erythrocyte metabolic reprogramming due to S1P [177]. Metabolomic studies of RBCs have highlighted consistent sphingolipid phenotypes of neurodegenerative diseases [178] and the role of elevated S1P in promoting sickle cell disease progression [179]. Because RBCs are the largest reservoir of circulating S1P [180,181] and ceramide formation by sphingomyelinase contributes to eryptosis [182], understanding RBC sphingolipid metabolism is essential to elucidate the contribution of RBC-derived S1P in the pathogenesis of various diseases [183].

### RBCs contribute to organismal homeostasis beyond oxygen transport activity

RBC-GEM includes all the known amino acid transporters and the metabolically active enzymes, elucidated through proteomics and supported by experimental evidence in the literature. The role of amino acids in erythrocyte metabolism was once thought to be limited to glutathione synthesis [184]; however, proteomic and metabolomic tracing experiments have revealed a diverse set of reactions in erythrocytes utilizing amino acids as substrates to drive metabolic processes. Erythrocytes contain metabolically active enzymes for arginine catabolism in nitric oxide regulation [185–188], transamination for glutamate homeostasis [27,189,190], and methionine salvage [4,33]. The available concentrations of amino acids may modulate the hypoxic response in erythrocytes [109,191]. Consequently, erythrocyte membranes have at least seven different amino acid transport systems that facilitate the rapid exchange of up to 17% of their total amino acid pool with surrounding plasma [192], highlighting their role as circulating reservoirs of amino acids and vitamins for maintaining organismal homeostasis and facilitating cross-talk between RBCs and other cell types [193–195].

We expanded NAD metabolism with NAD glycohydrolase (*CD38*) activity [53,196,197], dihydronicotinamide riboside (NRH) salvage via adenosine kinase (*ADK*) [198], and oxidation through NRH:quinone oxidoreductase (*NQO2*) [199–201]. We also included *GAPDH* catalyzed and spontaneous generation of hydrated NADH [131], and subsequent repair through enzyme-catalyzed epimerase and ATP-dependent dehydration reactions, once stated to exist in RBCs [202] and later confirmed in approaches to elucidate the RBC proteome [27,28,54]. Erythrocytes have translocator protein 2 (*TSPO2*) [203], involved in cellular import of the heme precursor 5-Aminolevulinic acid [204], cytoplasmic enzymes of the heme biosynthetic pathway (*ALAD*, *HMBS*, *UROS*, *UROD*), and ATP-binding cassette sub-family member 6 (*ABCB6*), involved cellular efflux of porphyrins at the plasma membrane [205,206], forming a non-canonical pathway of unknown significance. Pantothenate and CoA metabolism [207,208], folate metabolism with its connections to AICAR metabolism [209,210], thiamine metabolism [211,212], and vitamin E recycling at the erythrocyte membrane were also included [73,213] in the RBC-GEM network.

RBCs have several other metabolic capabilities that are increasingly being recognized for their physiological and pharmacological relevance. They have been shown to have roles in the endocrine system [214], act as modulators of innate immunity [215,216], and hydrolyze bioactive peptides including Angiotensin II [217–219]. Therefore, we included catecholamines such as epinephrine, norepinephrine, and dopamine along with subsequent oxidation and glutathione conjugation reactions [75]. We also included various post-translational modifications (PTMs) and repair reactions including: deglycation by fructose 3-kinase (*FN3K*) [100,220] and related protein (*FN3KRP*) [221], methylation by methyltransferases including *PCMT1* [4], glycosylation by residual activity of blood group proteins [222,223], signaling through phosphorylation and dephosphorylation [29,224,225], protein degradation through ubiquitin-mediated proteolysis [93,226], and alkylation via transglutaminase (*TGM2*) [227]. The multitude of PTMs and repair reactions included in RBC-GEM are indicative of their importance for RBC metabolic reprogramming, adaptivity, and timing of senescence. PTMs diversify protein functions; therefore, understanding PTMs is important to discovering the full functionality of the RBC proteome.

### Classification of membrane transport proteins

Understanding the influence of genetic variation on RBC membrane protein expression is important to understanding pathological metabolic states, exemplified by the formal recognition of several membrane transport proteins as ‘blood group’ proteins due to their immunological and pharmacological significance [228,229]. All transport reactions in the RBC network were grouped within a single representative subsystem. We classified transport reactions using the Transport Classification System [115] to assign classification numbers to transport proteins and their associated reactions (Fig 4B). The “Unclassified” label was assigned to reactions representing passive transport via diffusion in addition to reactions associated with unknown or unclassified transport proteins (Table K in S2 File). Classification of transport proteins and associated reactions revealed that less than 3% of transport reactions in RBC-GEM involve the transport of ions and small molecules through membrane pores and channels. Despite being responsible for a small percentage of reactions, these transport proteins have critical importance for erythrocyte osmotic regulation and gas transport functions; they serve as a nexus for sensing electrical, chemical, and mechanical changes [230–237], dictating the RBC metabolic response accordingly. Proteins in this category include the blood group antigens Piezo-type mechanosensitive ion channel component 1 (*PIEZO1*), Rhesus complex proteins (*RHAG*, *RHCE*, *RHD*) [233], Aquaporins 1 and 3 (*AQP1*, *AQP3*) [235,238] the urea transporter (*SLC14A1*), as well as Ca^2+^ channel and Ca^2+^-regulated ion channel proteins such as the Gardos channel (*KCNN4*) [231].

The largest class of transporters, both in terms of total number of proteins and in associated transport reaction, was the class of electrochemical potential-driven gradient transporters, with nearly 40% of transport related genes responsible for 35% of the reactions. Most reactions represent the different mechanisms of amino acid transport in the RBC; at least seven different amino acid transport systems have been discovered, with unclear metabolic functions in RBCs other than providing the precursors for glutathione synthesis [184]. Further compelling evidence supports the role RBCs play in interorgan amino acid delivery tissues. The numerous routes for amino acid exchange further highlight the contribution of RBCs toward maintaining metabolic homeostasis outside of their gas transport functions [192,193,195,239,240]. The remainder of reactions reflect carbohydrate uptake [241], monocarboxylate exchange [242], folate exchange [243], facilitated diffusion of nucleotides [136,244–246], ion cotransport and counter-transport [236], and numerous anion exchange reactions facilitated by the Band 3 anion exchanger (*SLC4A1*) [247].

Most reactions catalyzed by primary active transporters are associated with the ATP-dependent efflux of a broad selection of metabolites, including, but not limited to, phospholipids, porphyrins, xenobiotics, oxidized glutathione, glutathione-conjugated steroids and catecholamines, and lipid peroxidation products. Aside from their broad specificity, the ATP-binding cassette (ABC) transporters form the genetic basis for several known blood groups (*ABCB6* [248], *ABCC1* [249], *ABCC4* [250], *ABCG2* [251]). Conversely, the P-type ATPases in erythrocytes are responsible for the efflux of a small selection of substrates and have significant roles in normal erythrocyte function. The protein complexes within this group catalyze Na^+^/K^+^-ATPase, and Ca^2+^-ATPase activities in the erythrocyte membrane, respectively consuming 40% and 10% of the total ATP produced by erythrocytes to maintain volume homeostasis and the electrochemical gradient across the plasma membrane. Working in concert with the ABC-transporters that “flop” PC, the P4-type ATPases “flip” externalized PE and PS to prolong the overall loss of plasma membrane asymmetry, a key signal for senescence in erythrocytes [65,252]. Erythrocytes also demonstrate V-ATPase activity [253] with recent proteomics suggesting a contributing role in membrane damage through acidification of the extracellular environment of cold-stored RBCs [94].

The smallest classification group of transporters corresponds to electron transport across the plasma membrane. Transmembrane electron transport is facilitated by ascorbate-dependent reductase (*CYBRD1*), responsible for vitamin C homeostasis at the erythrocyte membrane after the maturational loss of the Na^+^-dependent vitamin C transporter [213,254,255]. It should be noted that ferrireductase (*STEAP3*) also belongs to the same class; however, it is primarily an endosomal protein involved in transferrin-mediated iron uptake [256,257]. *STEAP3* activity has been measured in mature murine RBCs, whereby it contributes to the reduction of free ferric iron to the reduced ferrous state. In so doing, *STEAP3* might promote Fenton chemistry by recycling a rate-limiting substrate for the generation of reactive oxygen species [256].The radical species resulting from excess *STEAP3* activity have been linked to elevated lipid peroxidation in mice and humans, a phenomenon associated with RBC vesiculation, splenic sequestration, and extravascular hemolysis. Interestingly, the erythrocyte contains a few proteins with incompletely characterized transport systems, including the non-ABC multidrug exporter, RalA-binding protein 1 (*RALBP1*) [74], the Ca^2+^-dependent phospholipid scramblase (*PLSCR1*) [258], and *TSPO2*, recently shown to mediate 5-aminolevulinic acid uptake [203,204] and VDAC-mediated ATP export [203].

### Proteome-constrained modeling of RBC metabolism

Proteome-constrained modeling of GEMs has previously proven to be a simple, yet effective tool for understanding cellular metabolism [259]. Proteome-constrained models do not require numerous uptake and secretion measurements to accurately simulate phenotypes. In addition, they are particularly suitable in facilitating predictions of overflow metabolism that necessitate physiological trade-offs from having insufficient enzyme concentration and/or catalytic capacity in the presence of excess substrate. Furthermore, the explicit representation of proteins in the mathematical problem allows for quantitative prediction of protein abundance.

**Fig 5:**
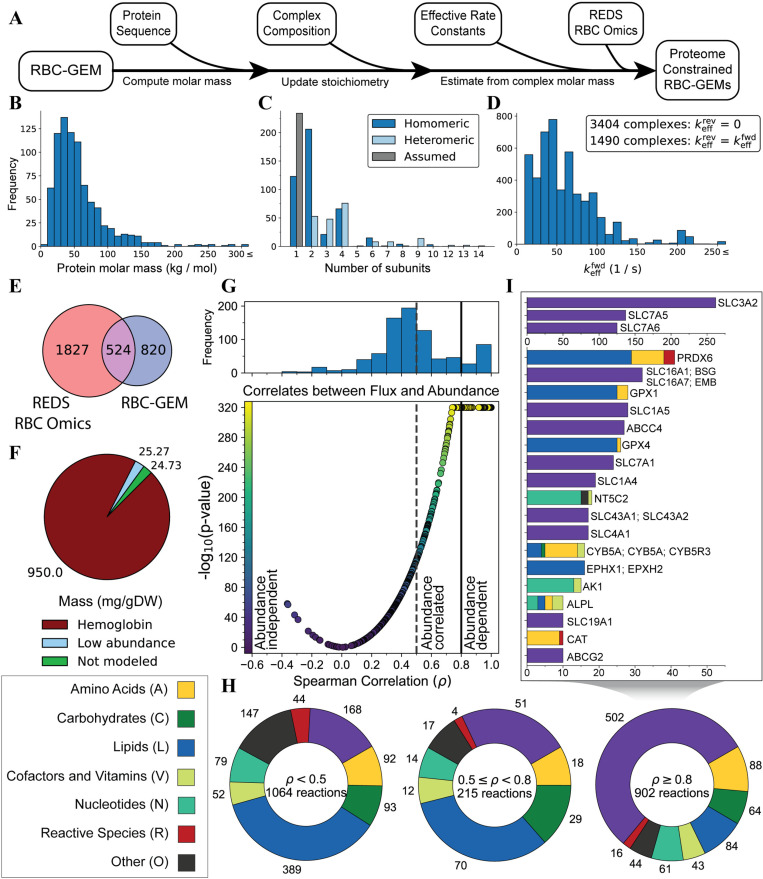
Proteome-constrained modeling of RBCs using REDS RBC Omics proteomic data. (A) Workflow to formulate a proteome-constrained RBC-GEM model. Distributions of (B) molar mass calculated from protein sequence, (C) complex subunit composition, and (D) effective forward rate constants used to form the proteome-constrained RBC-GEM. (E) REDS RBC Omics data mapped to 524 proteins in RBC-GEM, (F) representing approximately 97.5% of the normalized proteome by mass. (G) Correlates between maximum reaction flux and associated enzyme abundance, determined through flux variability analysis on the 1848 context-specific models derived from REDS RBC Omics data. (H) Metabolic categories of reactions classified as abundance-dependent, abundance-correlated, or abundance-independent. (I) Proteins associated with at least ten abundance-dependent reactions. Proteins were grouped if associated reaction sets were identical.

To formulate a proteome-constrained model of RBC metabolism, we utilized the annotated RBC-GEM 1.2.0 reconstruction to extract protein sequences, oligomeric protein structures, and complex compositions from the UniProtKB [43], Reactome [260], HumanCyc [261,262], and ComplexPortal [263] databases. We implemented proteomic constraints following OVERLAY computational pipeline for proteome-constrained modeling, initially estimating effective rate constants based on complex molar mass and scaling them according to solvent accessible surface area (SASA), as in previous studies [259,264,265]. The baseline proteome-constrained RBC-GEM reconstruction contained 820 distinct proteins forming a total of 887 complexes that map to 2181 ‘enzyme’ entities through 6384 non-zero effective rate constants (Fig 5).

We mapped proteomic data from the REDS RBC Omics project [100] onto the baseline proteome-constrained model to formulate context-specific models for further analyses. Previously, blood donors that ranked in the 5^th^ or 95^th^ percentile for hemolytic propensity in the REDS-III study [266] were invited to donate an additional unit in the “recall phase” of the study. As part of the REDS-IV-Pediatrics project [267], blood was collected from 643 donors and subsequently stored for 10, 23, and 42 days before high-throughput proteomic analyses. We collected relatively quantified proteomic data for 616 donors whose measurements were available at all three time points. We applied the “total protein approach” [28,268] to compute protein copy numbers by normalizing samples to the hemoglobin concentration of the corresponding donor measured at time of donation. Absolute abundance values of estimated proteins concentrations (Table D in S6 File) and copy numbers (Table E in S6 File) were quantified across 1848 samples. Across the 1848 samples obtained from the RBC Omics data, a total of 1827 proteins were identified and associated with unique UniProtKB identifiers. For each sample, the measurements for hemoglobin proteins were scaled to 95% of the protein content, with the remaining proteome scaled to the remaining 5%. Of the 1827 proteins, 524 mapped to proteins represented in the RBC-GEM metabolic network (Fig 5E), corresponding to an average of 97.527% of the proteome by RBC dry mass (Fig 5F).

For each sample, we computed the best-fit proteome to derive 1854 context-specific models representing the RBCs of the 616 donors after 10, 23, and 42 days of storage with an additional six models representing the mean and median protein abundances. We computed the corresponding fluxome for each context-specific model using proteome-constrained FVA [259], with the Na^+^/K^+^-ATPase set as objective reaction and constrained to 0%, 50%, 90% and 99% of its maximum value. We computed the Spearman rank correlation coefficient (*ρ*) between the maximum reaction flux and the total abundance of its associated catalytic complexes for all enzymatic reactions to classify reactions based on their abundance dependence (Fig 5G). Out of the 2,181 enzymatic reactions in the RBC-GEM reconstruction, 902 reactions were classified as abundance-dependent (0.8 ≥ *ρ*) 215 reactions as abundance-correlated (0.5 ≤ *ρ* < 0.8), and 497 reactions as abundance-independent (*ρ* < 0.5). An additional 567 enzymatic reactions were classified as abundance-independent due their inability to carry any flux (Table G in S6 File).

Approximately 55% of abundance-dependent reactions were associated with transport functions (Fig 5H). Many of these reactions were associated with the seven distinct amino acid transport systems known to exist in RBC membranes (Fig 5I). Each amino acid transport system has its distinct substrate preferences [71,184]; thus, these results suggest genetic factors affecting expression of amino acid transporters contribute to observed metabolic differences in mature RBCs [68,92,269]. Other abundance-dependent reactions corresponded to ROS and lipid peroxide detoxification reactions, catalyzed by glutathione peroxidases (*GPX1*, *GPX4, PRDX6*), and ATP-dependent export reactions, catalyzed by proteins for responsible for the PEL and JR blood groups (*ABCC4* [250] and *ABCG2* [251], respectively). Many abundance-correlated and abundance-independent reactions were involved in lipid metabolism (Fig 5H). However, the degradation of key proteins in lipid synthetic and elongation pathways prevent these reactions from carrying flux in the mature erythrocyte, regardless of the expression of other proteins in the pathway (e.g., *FASN*). There were also many abundance-dependent reactions that had only one or two enzymes responsible for catalyzing the reaction. Some of these enzymes (i.e., *HK1*, *G6PD*, *PFKP*, *CD38*, *PKLR*, *SMOX*). have been previously identified by metabolite Quantitative Trait Loci (mQTL) analyses as potential genetic factors influencing the quality of stored RBCs [68,100,270], making them prospective targets for further exploration and experimentation.

**Fig 6:**
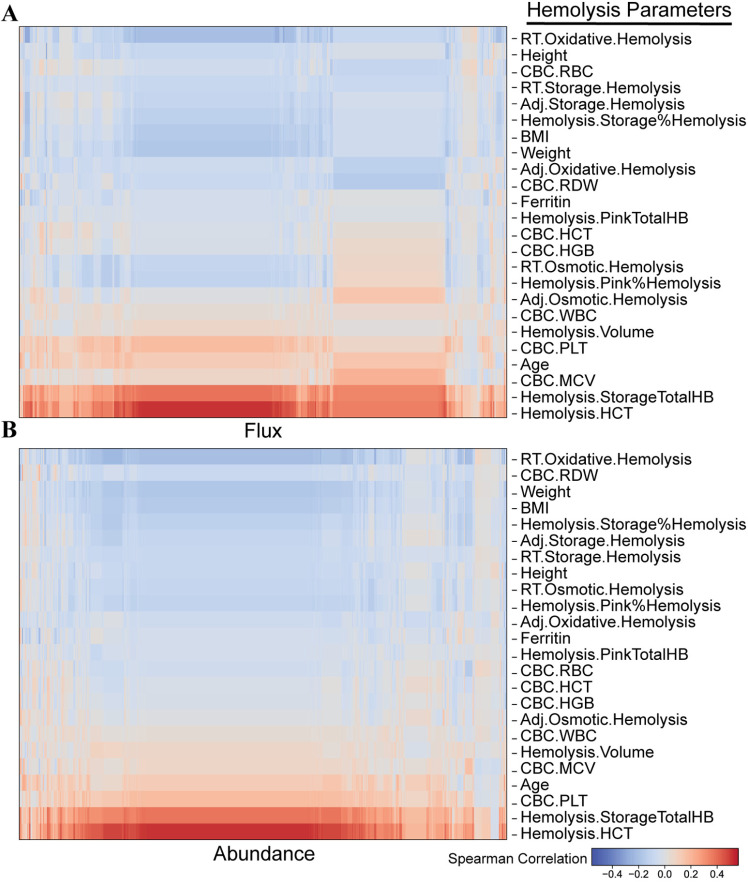
Correlations with donor metadata of REDS RBC Omics. Spearman correlations were computed between (A) hemolytic parameters and maximum flux, and (B) hemolytic parameters and enzymatic abundance. Results are clustered using Euclidean distance to enhance visual clarity.

We also computed correlations with the donor metadata with the maximum reaction flux (Fig 6A and Table H in S6 File) and enzymatic abundance associated with reactions (Fig 6B and Table I in S6 File). The strongest positive correlations with both maximum flux and enzymatic abundance were found with hemolysis parameters for hematocrit and total hemoglobin content of stored blood units. Conversely, the strongest negative correlations were observed to be associated with oxidative hemolysis of the recall results from the transfer bags used in the study. These results suggest that factors positively influencing hemolysis parameters related to hemoglobin content inversely influence oxidative hemolysis; however, additional experimentation and analyses are required to determine the precise nature of the relationships between these variables.

It requires significant computational resources to repeatedly simulate hundreds of models; thus, we utilize the results from the flux variability analyses to formulate a representative proteome-constrained RBC-GEM model. Constraint bounds for reaction flux and protein abundance were set in the representative model using the effective minimum and maximum values across all the context-specific models (Table L in S6 File). The proteome-constrained RBC-GEM, representative of the 1848 context-specific REDS RBC Omics, is made available as an SBML file (S7 File).

## Discussion

RBC-GEM 1.2.0 is the most expansive and comprehensive reconstruction of RBC metabolism to date, supported by 29 proteomic datasets and a bibliome of 1000+ RBC-specific publications representing over 75 years of available research on the human RBC. Biochemical pathways are visualized in the entire RBC metabolic network through a global metabolic network map, generated by the Escher visualization tool. We provide an interactive version of the map, allowing for visual contextualization of different user-defined data. Connectivity analysis demonstrated the RBC metabolic network was not inherently ‘fragmented’ and that understanding RBCs through various omics approaches have revealed a richer, more connected metabolic network than previously known. By using high-throughput multi-omic data, a comprehensive understanding of the catalytically active RBC proteome is also achievable [30,31,111]. Furthermore, coalescence of information about pathological metabolic states in RBCs illuminated the physiological significance of ‘moonlighting’ functions of enzymes and previously overlooked metabolic pathways.

Typically, proteins with larger copy numbers are more likely to carry meaningful flux; however, the human RBCs contains various exceptions that were necessary to consider when building the RBC-GEM reconstruction. For example, absolute quantitative proteomic data of mature healthy human erythrocytes reveal fatty acid synthase (*FASN*) is present in relatively moderate abundance [28]. Tracing experiments demonstrate that the enzyme is catalytically active if the Malonyl-CoA is present in the RBC environment; however, the lack of Acetyl-CoA carboxylase in mature healthy erythrocytes prevents Malonyl-CoA from forming [167]. Additionally, proteins that are historically known to exist and demonstrate activity in the RBC, such as hexokinase (*HK1)* and pyrimidine 5’-nucleotidase 1 (*NT5C3A*), show up in less than half of proteomic datasets. Even within a modern proteomic dataset identifying more than 2600 RBC proteins [28], *HK1* was not detected. We envisioned the RBC-GEM being able to accommodate a wide variety of modeling scenarios for biological processes across many different timescales, and we anticipate that users will derive their own RBC models using RBC-GEM as a reference. Because high variability exists within proteins identified as part of the RBC proteomic studies (S1 Fig), our approach to the reconstruction involved including all proteins we found supported through proteomic and experimental evidence, and by adding catalytically possible reactions. As an example, *FASN* and its associated catalytic activity were included in the reconstruction, with the lack of Acetyl-CoA carboxylase in the reconstruction preventing the pathway activity from carrying any meaningful flux in healthy cells. Consequently, FVA calculations were essential in determining the effective flux bounds that define the feasible solution space of the reconstruction.

We implemented proteomic constraints based on the OVERLAY computational pipeline for proteome-constrained modeling [259]. Integration of kinetic constants obtained from biochemical assays and computational estimations often result in over-constrained models, requiring iterative approaches to relax constraints, prevent infeasible solutions, and eventually become comparable to experimental observations [271–273]. However, the OVERLAY framework avoids over-constraining models through simplifying assumptions for the initial estimates of rate constants and the unification of complexes under a single ‘enzyme’ entity in the constraint formulation. Furthermore, catalytic properties are integrated into the OVERLAY formulation model in the form of an effective turnover number, which may be further calibrated using available omics data for additional consistency with observed phenotypes.

There are several possible sources of uncertainty that must be considered when modeling the RBC proteome due to its unique composition. Because the protein content of RBCs is primarily hemoglobin, strategies to obtain deep coverage of the low abundance RBC proteome often require depleting the hemoglobin first [27, 28] for increased insight. Furthermore, absence of proteomic evidence for a known RBC protein in the low-abundance proteome may be caused by technical and methodological reasons, as strongly indicated by the variability in the RBC proteome across studies (S1 Fig). Thus, we defined a “relaxation” proteome budget to represent a variable pool of unspecified protein. After fitting representative mean and median models, we relaxed the proteome constraints according to the size of the unspecified protein pool via the slack term (S2 Fig). Through this implementation of constraint relaxation, we ensured feasible solutions were obtainable for all context-specific models, accounted for variability in the proteomic data, and represented uncertainty within the modeling workflow in an unbiased fashion.

The use of simplifying assumptions for parameter reconciliation necessitates caution when interpreting results numerically. Modeling isozymes within a cellular context often comes with various difficulties and limitations due to the inability to ascertain the relative load of each isozyme. Isozymes may also differ in catalytic and regulatory properties, which is further complicated by heterogenous subunit oligomerization, as illustrated with the PFK platelet, muscle, and liver subunits [274,275]. As these problems specific to isozymes are in addition to the previously stated need for reconciliation of *in vitro*, simplifying assumptions become necessary and results must be interpreted within the context of those assumptions. Both dynamic modeling and proteome-constrained modeling necessitate the need for different experimental data types to form predictions. Thus, future modeling efforts should be focused on incorporating additional experimental measurements to increase model performance and refine its predictive capabilities.

One critical datatype relevant for improvement of model performance would be the incorporation and refinement of kinetic constants. The use of high-throughput omics enables the estimation of the “kinetome,” defined as the collection of cellular enzymatic rate constants [276,277]. There are multiple sources from which kinetic constants can be derived, especially with regards to human RBCs. There is an abundance of available *in vitro* kinetic data throughout the experimental literature for human RBCs, a consequence of their relative metabolic simplicity with respect to nucleated cell types, accessibility as a human cell-type, and significant past role in advancing development of whole-cell biophysical models [278]. Several machine learning-based methodologies have been developed to predict *in vivo* kinetic constants from *in vitro* kinetics and multi-omics data, taking into consideration organismal information, amino acid protein sequence, pH, and/or thermodynamic properties [279–281]. Metabolomic data may be mapped onto the reconstruction to calibrate the set of rate constants that fit the data accordingly.

The proteome-constrained model formulation is also optimal for quantitative exploration and interpretation of proteomic data [282]. Proteomics-derived *in vivo* maximal rates provide a physiologically relevant approach for the collection of enzyme parameters. The obtained values can then be used to parameterize dynamic models with explicit mechanistic representations of enzymes for insight into their catalytic and regulatory properties [283,284]. The RBC lacks the machinery necessary to synthesize new proteins *de novo*; consequently, the RBC proteome loses functionality over time, affecting essential functions in gas transport. With high-quality quantitative proteomic data of pure RBC populations, computational methods for estimation of *in vivo* rate constants and proteome-constrained flux states, and tools for dynamic characterization of the proteome, it is possible to define the functional capacity of the low abundance RBC proteome to obtain physiologically meaningful insights into the underlying mechanisms governing clinical phenotypes observed in translational medicine.

RBC-GEM is available in a version-controlled GitHub repository (https://github.com/z-haiman/RBC-GEM) where the versions of the reconstruction are hosted as fully annotated SBML file (S1 File) and as flat table files. A proteome-constrained RBC-GEM, representative of the 1848 context-specific REDS RBC Omics, is also made available as an SBML file (S7 File). Through our implementation of a version-controlled framework, we adhere to principles of data stewardship and ensure that RBC-GEM remains a curated, high-quality knowledge base, consistent with current knowledge and community consensus, as improvements are continually made in a tractable and traceable manner. RBC-GEM 1.2.0 represents a critically important step toward the development of a proteomically complete reconstruction of RBC metabolism. This reconstruction paves the way for the development of the next generation of RBC whole-cell models.

## Methods

### Preparing iAB-RBC-283 for expansion

RBC-GEM was constructed using the iAB-RBC-283 [13] as the initial starting point from which further refinements and expansions were made using Human-GEM [11] and RBC specific literature. The iAB-RBC-283 reconstruction was downloaded from the BiGG Database version 1.6 [41,44], and identifiers were harmonized with the current iteration of the Human-GEM (version 1.19.0 [39]), downloaded from the MetabolicAtlas [11], to assist in providing a common framework to work with both reconstructions.

We addressed the representation of lipids in the RBC metabolic network. Fatty acid chains in iAB-RBC-283 were not represented as generic R-groups, as in other human reconstructions, but instead as three individual lipid species. To accommodate for the increased number of lipid species represented in RBC-GEM, we utilized R-groups to represent lipid species like the Human-GEM reconstruction, resulting in the replacement of 125 reactions (Table L in S2 File) and 62 metabolites (Table M in S2 File) in iAB-RBC-283 with 18 pooled reactions and 10 representative species utilizing R-groups. We condensed the carnitine shuttle reactions from three pairs of irreversible reactions with opposite directions into three individual reversible reactions representing the net reaction, justified by the known reversibility of the carnitine palmitoyltransferase enzyme in RBCs [61]. We also removed 11 intracellular sink and demand reactions to prevent their interference in the identification of blocked reactions and potential areas of reconstruction expansion.

iAB-RBC-283 contained 346 protein products, including alternate splice variants, represented by 283 distinct genes. We simplified the gene-protein-reaction associations through the removal of splice variants and associated each gene with both its official gene symbol [45] and UniProtKB identifier, resulting in 283 unique gene entries. The resulting reconstruction, dubbed RBC-GEM 0.3.0, was hereafter treated as the main draft reconstruction for subsequent refinement and expansion.

### Collection of proteomic data

Published proteomic data for erythrocytes was aggregated from 29 datasets [19–21,27–29,39,54, 80–100] spanning 20 years of RBC proteomic research to facilitate the expansion of the RBC network. iAB-RBC-283 was derived through the integration of three proteomic datasets [19–21] with the global human metabolic reconstruction at the time, Recon1 [14]; however, protein databases have since undergone significant changes, including the discontinuation of the International Protein Index (IPI) database in favor of UniProtKB [104]. Studies utilizing either IPI [19,20,81,82] or GenInfo (GI) identifiers [80,83,84,86] were mapped to UniProtKB identifiers. The consolidated list of UniProtKB identifiers was run through the UniProtKB ID mapping service [43], ensuring they reflected updated protein entries. Proteins that were not successfully mapped to the UniProtKB database were confirmed to be obsolete. In total, over 4000 proteins were detected at least once across 20+ different RBC specific proteomic datasets (Table B in S2 File and S1 Fig).

### Reassessment of the existing reconstruction

Using the COBRApy python software package (v0.29.0 [285,286]), the complete list of 4000+ proteins was first mapped onto the initial RBC-GEM. Genes and biochemicals without proteomic evidence were then subjected to additional scrutiny through targeted literature searches to justify their presence or removal from the reconstruction. Consequently, genes and associated reactions responsible for initial steps of the Kennedy pathway for de novo lipid synthesis were removed because studies demonstrate erythrocytes lack the phosphotransferase enzymes [169], made further evident by the accumulation of cytidine phosphodiester compounds in patients with pyrimidine nucleotidase deficiency [287]. We also removed the genes and reactions associated with the final steps of heme synthesis, as they are known to be localized to the mitochondria. However, steps leading up mitochondrial transport were kept, as recent studies demonstrate erythrocytes contain *TSPO2* [203] involved in cellular import of the heme precursor 5-Aminolevulinic acid [204], and *ABCB6* involved cellular efflux of porphyrins at the plasma membrane [205,206]. Enzymatic heme degradation was also removed from the reconstruction because evidence demonstrated non-enzymatic heme degradation by reactive species as the primary route [155]. However, the abundant *BLVRB* was kept for its physiologically important role as the NADPH flavin reductase for methemoglobin reduction in RBCs [288,289]. RBCs lack the capacity for glucuronidation [290] and do not exhibit significant glycerol kinase activity [291]. RBCs have also been shown incapable of significant alpha-glutamyl dipeptide transport [292–295] and do not have 5-oxoprolinase activity [107]. Subsequently, we removed the reactions and associated genes enabling these capabilities within the RBC-GEM reconstruction.

The curation process, combined with the additional proteomic evidence, led to the replacement of 18 biochemical and transport reactions in favor of analogous reactions with better evidentiary support. Except for mitochondrial enzymes, such as coproporphyrinogen-III oxidase (*CPOX*), protoporphyrinogen oxidase (*PPOX*), and ferrochelatase (*FECH*), most removed proteins were due to an inability to find direct supporting evidence for their inclusion. We removed these genes from the reconstruction and note that additional evidence may call for their reinclusion in a future iteration of RBC-GEM. In summary, 36 distinct biochemical reactions (Table L in S2 File), eight metabolites (Table M in S2 File), and 113 genes (Table N in S2 File) were removed from the RBC-GEM reconstruction.

### Expansion of the RBC-GEM

We mapped the complete list of 4000+ proteins to the Human-GEM reconstruction to identify candidate reactions to add to the RBC-GEM [41,44]. After filtering out the reactions occurring within organelles, we explored the remaining candidate reactions in a subsystem-dependent manner. For each subsystem, we started with reactions associated with proteins that appear in multiple proteomic datasets and searched the literature for supporting evidence. Often, we were able to find supporting evidence in the form of human RBC-specific literature; however, we also relied on biochemical databases (e.g., KEGG [42], UniProtKB [43], RHEA [296]), pharmacological databases [297], and literary sources pertaining to other human cell-RBC of other mammalian species. We also relied on literature about pathological states to verify typically inactive or non-canonical pathways in RBCs. For example, pyrimidine 5’-nucleotidase deficiency [140] provided insight into deoxynucleotide detoxification. As a cell without protein synthesis capabilities, the presence and relevance of enzymatic activity in RBCs is often subject for debate. Consequently, we included all evidence utilized in the curation process for the addition of genes (Table D in S2 File) and reactions (Table F in S2 File).

Erythrocytes are constantly exposed to ROS from external and internal sources of oxidants [51]; thus, we assumed that spontaneous oxidation events due to reactive species are more likely to occur than other types of reactions. Additionally, hemoglobin has been shown to participate in a variety of reactions outside of the standard role it has in gas transport [52,150–153]. The degradation of hemoglobin is non-enzymatic, generating reactive species [155] before glutathione facilitates degradation of hemin, as evident in hemolytic disorders like beta thalassemia [156,157]. Hemoglobin is the most abundant protein in red cells, we therefore assumed that reactions shown to be catalyzed by hemoglobin *in vitro* were also possible *in vivo* [52,150–153]. The inclusion of several reactions involving the formation and detoxification of reactive oxygen, nitrogen, and sulfur species, especially in the context of their interactions with hemoglobin, led to the formation of the “Reactive Species’‘ category [154].

### Curation of subsystem categories

Assignment of reactions to metabolic subsystems can provide meaningful biological context within a larger network analysis, aiding in their analysis and visualization [56]. We assign subsystems following conventions used in the Human-GEM [11]. The subsystems were then compared to KEGG database [42] to broadly group subsystems into the general categories: “Amino acid metabolism,” “Carbohydrate metabolism,” “Lipid metabolism,” “Metabolism of cofactors and vitamins,” and “Nucleotide metabolism.” Subsystems pertaining to both hemoglobin and spontaneously formed reactive species were categorized as “Reactive species.” All other subsystems, such as those pertaining to PTMs of proteins or peptide metabolism, were categorized as “Other” for clarity in visualization (Table J in S2 File).

### Visualization of the global RBC metabolic network

In tandem with the development of RBC-GEM, we developed a network map of the full erythrocyte metabolic network using the Escher Network visualization tool [55]. In the construction of the map, we grouped reactions within the context of their subsystems and subsequently color-coded reactions based on their generalized category. We focused our initial efforts on the development of a global metabolic map of erythrocyte metabolism so that users of RBC-GEM could contextualize relevant information within the full network context before tailoring the reconstruction for specific applications. The map is provided as an Escher JSON file, enabling users to apply it for their own purposes, including the derivation of new pathway visualizations via Escher without having to start from a blank canvas. The metabolic map is also provided in standard SBGN and SBML layouts, generated by EscherConverter tool (version 1.2), for accessibility and interoperability with other network visualization tools. All map files are found in the supplement (S4 File).

### Functional testing of metabolic capabilities

Throughout each iteration of the reconstruction processes, the COBRApy implementation of flux variability analysis (FVA) [298–300] was utilized to calculate the minimum and maximum allowable flux through each reaction. Reactions with non-zero flux values may be activated under specific conditions, indicating that they may have relevant physiological functions. For reactions producing a zero flux, literature was consulted to determine whether the associated pathways should be functional in the erythrocyte. As an example, erythrocytes have demonstrated capability to synthesize and elongate lipids so long as Malonyl-CoA is present in the environment; it is the inability for erythrocytes to produce Malonyl-CoA at a non-negligible rate that prevents activation of the pathway. Consequently, all reactions that stem from Malonyl-CoA produce a zero flux in FVA simulations. Another example can be seen in the treatment of the acyl-carnitines throughout the reconstruction. Acyl carnitines serve as markers of membrane integrity in erythrocytes [78,93,301,302]; however, the maintenance of the pool involves the reversible transfer of the acyl group between carnitine and CoA. These reactions require additional pseudoreactions to produce non-zero fluxes in FVA simulations. We opted to keep these reactions and metabolites in reconstruction regardless of the flux value produced by FVA, empowering users of RBC-GEM to make their own decisions regarding relevance and inclusion in their applications.

### MIRIAM compliance and MEMOTE standardized testing

Recon3D [10] was downloaded from the BiGG Database version 1.6 [41,44] and used throughout the reconstruction process to map between BiGG and MetabolicAtlas identifier namespaces. Newly added reactions and metabolites that were unique to RBC-GEM were given new identifiers following BiGG ID standards. Reactions and metabolites previously assigned ambiguous or non-descriptive BiGG identifiers were also assigned new identifiers using BIGG standards following the retirement of their original ID. New reactions were mass balanced using the ChemAxon Chemicalize application [303] to compute the molecular formula and charge of associated metabolites at pH 7.25. The species representative of protein residues and hemoglobin subunits in RBC-GEM included generic R-groups in their formula to represent the remainder of the macromolecule after mass and charge-balancing associated reactions.

Through the MetabolicAtlas, we enriched metabolites and reactions with the connections to external resources originally contained within the Human-GEM. Rather than adhere to the use of Ensembl identifiers as seen in Human-GEM, we opted for gene identifiers based on the HUGO Gene Nomenclature Committee (HGNC) database, as we felt they were human readable, short and memorable, and SBML compliant in most circumstances. Each gene/protein contained in RBC-GEM was uniquely identified by its gene symbol [45] and set to represent one reviewed and current entry in the UniProtKB database [43]. We utilized the UniProtKB ID mapping service to extract external identifiers connecting gene information across multiple resources and annotated RBC-GEM accordingly. Compact identifiers for over 60 different databases could be extracted and mapped into the RBC-GEM, enriching protein annotations in the reconstruction. We also mapped the proteins in RBC-GEM to other databases for pharmaceutical and genetic information, including drugs found in DrugBank [46] (Table G in S2 File), phenotypes in OMIM [48] (Table H in S2 File), and SNPs found in both the UniProtKB [43] and NCBI SNP databases [47] (Table I in S2 File). All annotations adhere to the guidelines for minimum information requested in the annotation of biochemical models (MIRIAM) [38,49] using compact identifiers that were resolved through Identifiers.org [50].

MEMOTE (version 0.17.0) was used to highlight changes made and identify knowledge gaps throughout the reconstruction processes. We configured the version-controlled GitHub repository to run the MEMOTE standardized test suite with the use of GitHub Actions at each main deployment. Through the combination of MEMOTE and GitHub, we ensured RBC-GEM was distributed within a framework for continuous integration of updates and delivery of the latest, quality-controlled version of RBC-GEM.

### Calculation of metabolite and gene connectivity

The stoichiometric matrix of RBC-GEM was used to compute the metabolite connectivity for each metabolite, defined as the total number of reactions in which the metabolite participates. After removing all pseudoreactions representing system boundaries and the pooling of lipid species, the connectivity of each metabolite was calculated through summation of the representative row in the stoichiometric matrix. Metabolite connectivity was determined with and without partitioning species by compartments. Resulting connectivity was rank ordered, forming a discrete distribution for subsequent comparison to the calculated connectivity distribution of iAB-RBC-283.

Metabolite connectivity analyses were first performed without any prior modifications to biochemical and transport reactions of the RBC metabolite network, providing an unbiased assessment of network topology. We subsequently pruned the networks for additional insight into the functional pathways. Networks were modified by removing metabolites representing water, protons, and metal and co-transport ions. Reactions were also modified by removing metabolites associated with substrate-product motifs for common redox pairs and ATP hydrolysis coupling (Table C in S5 File). Metabolite connectivity was calculated for the pruned RBC-GEM and iAB-RBC-283 reconstructions and compared. The gene connectivity was defined as the total number of reactions associated with the gene. The number of reactions per each gene in the unmodified RBC-GEM reconstruction was summed and the results were subsequently rank ordered.

### Proteome-constrained modeling of RBC metabolism

To construct a proteome-constrained model of RBC metabolism, we utilized the annotated RBC-GEM reconstruction to extract protein sequences, oligomeric structures, and complex compositions from the UniProtKB [43], Reactome [260], HumanCyc [261,262], and ComplexPortal [263] databases. Proteomic constraints were formulated following the OVERLAY computational pipeline for proteome-constrained modeling [259]. Our proteome-constrained model is formulated as the following optimization problem:


$$\begin{array}{l} \begin{array}{c} \underset{v,p,x,e}{\text{maximize}}{\text{c}}^{\text{T}}\text{v} \end{array} \end{array}$$ (1)

$$\begin{array}{c} S\cdot v=0 \end{array}$$ (2)

$$\begin{array}{c} v_{\text{lb}}\leq v_{i}\leq v_{\text{ub}},\forall i \end{array}$$ (3)

$$\begin{array}{c} 0\leq p_{j}\leq p_{\text{ub}},\forall j \end{array}$$ (4)

$$\begin{array}{c} {\text{p}}^{\text{T}}w\leq P_{\text{total}}\leq 1000 \end{array}$$ (5)

$$\begin{array}{c} C\cdot x\leq p \end{array}$$ (6)

$$\begin{array}{c} e={\text{e}}^{\text{fwd}}+{\text{e}}^{\text{rev}}=B\cdot diag\left(r\right)\;\cdot \;x \end{array}$$ (7)

$$\begin{array}{c} -k_{i,m}^{\text{rev}}e_{m}^{\text{rev}}\leq v_{i}\leq k_{i,m}^{\text{fwd}}e_{m}^{\text{fwd}},\;\;\;\;\;\;\forall i,m\left(i=m\right) \end{array}$$ (8)

$$\begin{array}{c} r_{l,m}^{\text{fwd}}=\frac{k_{l,\text{m}}^{\text{fwd}}}{k_{i,m}^{\text{fwd}}},\forall i,l,m\left(i=m\right) \end{array}$$ (9)

$$\begin{array}{c} r_{l,m}^{\text{rev}}=\frac{k_{l,m}^{\text{rev}}}{k_{i,m}^{\text{rev}}},\forall i,l,m\left(i=m\right) \end{array}$$ (10)

Through our adoption of the OVERLAY framework, we assumed the following:

1. The total amount of metabolic proteome may not exceed a weight fraction of the dry weight, referred to as the ‘proteome budget’.
2. Each annotated gene in the reconstruction corresponds to a unique protein. The molecular weight of each protein can be estimated by its amino acid sequence.
3. The rate constant of a certain catalytic complex is fixed regardless of reactions catalyzed, reducing the complexity of the optimization problem.
4. The concentration of an enzyme sets an upper limit on allowable reaction flux but does not force a reaction to carry flux.
5. The total abundance of proteins corresponding to the same gene product (e.g., isoforms with different sequences, proteins located in different compartments, etc.) are constrained to a constant pool. For species compartmentalization requirements in COBRApy [285] and SBML [304], all proteins are considered to belong to a single compartment.

Proteomic constraints were implemented by adding additional variables and constraints to the reconstruction as follows:

1. The proteome budget was represented by a pseudometabolite and a pseudoreaction that collectively constrain the total amount of protein (*P*_total_, mg/gDW) according to protein molar mass (**w**, mg/nmol). The total proteome budget was set such that it cannot exceed 1000 mg/gDW (=100% of cellular dry weight).
2. Protein dilution reactions were added to facilitate explicit representation of protein abundance (**p**, nmol/gDW) in the model. Protein concentrations must be non-negative.
3. Complex formation reactions were added to facilitate explicit representation of protein complex abundance (**x,** nmol/gDW) in the model. Each complex may not exceed the abundance of available protein subunit as determined by the complex stoichiometric composition (**C**).
4. Enzyme formation reactions were added to unite collection of indifferent protein complexes responsible for catalyzing a certain reaction. This unified entity is referred to as an ‘enzyme’ and exists as a pair representing forward (**e**^fwd^) and reverse (**e**^rev^) directions. The sum of forward and reverse ‘enzyme’ equals the total ‘enzyme’ abundance (**e**, nmol/gDW) associated with a reaction. Corresponding ‘enzyme’ dilution reactions were added to facilitate explicit representation of ‘enzyme’ concentration in the model.
5. Complex-to-enzyme mapping is facilitated by Boolean matrix (**B**) and the ratio (**r**) of effective rate constants (*k*, 1/hr) for each complex to the corresponding ‘enzyme’ entity.
6. Reaction fluxes (**v**, mmol/gDW/hr) are constrained by the effective rate constant of the ‘enzyme’ entity and its abundance value. The abundance value of the entity is representative of the associated complexes.

Previous computational studies revealed metabolic flux to be the most important predictor of catalytic rate. However, catalytic rate constants measured *in vitro* can vary greatly as they are strongly dependent on conditions used in the biochemical assay [271,305,306]. Furthermore, experimentally measured rate constants are not always available. Therefore, complex rate constants were initially estimated based on complex molar mass and scaled according to enzymatic surface area, as in previous studies [259,264,265]. The number of catalytic complexes active in the reverse direction were further reduced by setting zero values if the association reaction was considered irreversible. By assuming that rate constants are fixed for catalytic complexes, Equations (9) and (10) are simplified and initial rate constants can be approximated as follows:


$$\begin{array}{c} k_{i,m}^{\text{fwd}}=k_{i,m}^{\text{rev}}=k_{\text{avg}},\forall i,m\left(i=m\right) \end{array}$$ (11)

$$\begin{array}{c} k_{l,m}^{\text{fwd}}=k_{\text{avg}}{\left(\frac{x_{l}}{\frac{1}{N}\sum_{l=1}^{N}x_{l}}\right)}^{\frac{3}{4}},\forall l,m \end{array}$$ (12)

$$\begin{array}{c} k_{l,m}^{\text{rev}}= \{\begin{array}{cc} 0, & v_{i,\text{lb}}\geq 0\\ k_{\text{avg}}{\left(\frac{x_{l}}{\frac{1}{N}\sum_{l=1}^{N}x_{l}}\right)}^{\frac{3}{4}}, & v_{i,\text{lb}}<0 \end{array},\forall i,l,m\left(i=m\right) \end{array}$$ (13)

with the average effective rate constant (*k*_avg_) set as 65 s^-1^ (=2.34 * 10^5^ hr^-1^).

Estimates of RBC protein content can vary; however, nearly all agree that at least 90% of the RBC protein content is comprised of hemoglobin [20,22,27,307,308] with the remaining portion of the proteome considered to be the “low abundance” proteome. Thus, the proteome budget was partitioned into separate budgets for hemoglobin and the low abundance proteome as follows:


$$\begin{array}{c} P_{\text{HB}}=\underset{j\in \text{HB}\left(j\right)}{\sum }p_{j}w_{j} \end{array}$$ (14)

$$\begin{array}{c} P_{\text{LA}}=\underset{j\notin \text{HB}\left(j\right)}{\sum }p_{j}w_{j} \end{array}$$ (15)

$$\begin{array}{c} P_{\text{HB},\text{lb}}\leq P_{\text{HB}}\leq P_{\text{HB},\text{ub}} \end{array}$$ (16)

$$\begin{array}{c} 0\leq P_{\text{LA}}\leq P_{\text{LA},\text{ub}} \end{array}$$ (17)

$$\begin{array}{c} P_{\text{total}}=P_{\text{HB}}+P_{\text{LA}} \end{array}$$ (18)

where HB(*j*) represents the index set of hemoglobin proteins. The hemoglobin proteome budget (*P*_HB_) was set with a lower limit of 900 mg/gDW (=90% of protein content), while the low abundance proteome budget (*P*_LA_) was set with an upper limit of 100 mg/gDW (=10% of protein content. The full proteome-constrained RBC-GEM reconstruction contained 820 distinct proteins forming a total of 887 complexes that map to 2181 ‘enzyme’ entities through 6384 non-zero effective rate constants (S6 File).

### Preparation of RBC proteomic data

Proteomic data was previously collected and published as part of the Recipient Epidemiology and Donor Evaluation Study (REDS) RBC Omics project [100]. As part of the REDS-III project [266], blood was collected from 13,403 donors collected from across the United States and RBCs were tested for their hemolytic propensity after 42 days of storage. Donors that ranked in the 5^th^ or 95^th^ percentile for hemolysis were invited to donate an additional unit as part of a “recall phase” [309] for subsequent multi-omic analyses. Under the aegis of the REDS-IV-Pediatrics project [267], a total of 643 donors were successfully recalled, each providing an additional unit of blood which was stored for 10, 23, and 42 days prior to multi-omic analyses. Proteomic analyses were performed as previously described [68,93,100] on a total 1,929 samples representing RBCs from 643 donors on storage day 10, 23 and 42. Protein abundances were computed using the “total protein approach” [28,268]. Samples were normalized using the hemoglobin concentration measured at time of donation for the associated donor, and copy numbers were computed accordingly. Missing hemoglobin values were filled using the mean value across all other donors. Only donors with measurements available for all three time points were also considered. The final dataset contained absolute abundance values for 1848 samples, representing for 616 donors at three different time points. Absolute abundance values of estimated proteins concentrations and copy numbers quantified across all samples, as well as associated metadata, are found in the S6 File.

### Formulation of context-specific models for unbiased network analysis

To determine the best-fit protein vector, we formulated the following quadratic programming (QP) objective, subject to constraints previously defined in Equations (2–18):


$$\begin{array}{c} \underset{v,p,x,e}{\text{minimize}}{\left(diag\left(\text{c}\right)\left(\text{p}-{\text{p}}^{\text{data}}\right)\right)}^{\text{T}}\left(\text{p}-{\text{p}}^{\text{data}}\right) \end{array}$$ (19)

$$\begin{array}{c} c_{j}= \{\begin{array}{cc} \frac{1}{p_{j}^{\text{data}}}, & p_{j}^{\text{data}}>0\\ 1, & p_{j}^{\text{data}}=0\\ 0, & p_{j}^{\text{data}}=\text{NaN} \end{array},\forall j \end{array}$$ (20)

where the abundance values are obtained from proteomic data for the vector of the modeled proteins (**p**^data^). The weighting coefficients (**c**) are defined with weights increasing inversely to protein abundance, limiting the presence of unexpressed proteins in context-specific models. Weights for modeled proteins without abundance measurements are set to zero. The proteomic data was scaled to the upper limit of the associated proteome budget to satisfy the following:


$$\begin{array}{c} P_{\text{HB},\text{ub}}=\underset{j\in \text{HB}\left(j\right)}{\sum }p_{j}w_{j} \end{array}$$ (21)

$$\begin{array}{c} P_{\text{LA},\text{ub}}=\underset{j\notin \text{HB}\left(j\right)}{\sum }p_{j}w_{j} \end{array}$$ (22)

ensuring that proteomic data and modeled protein abundances were within the same magnitude and that the objective defined by Equation (19) could produce an excellent fit. Each sample was scaled such *P*_HB,ub_ = 950 mg/gDW and *P*_LA,ub_ = 50 mg/gDW, and the Gurobi Optimizer was used to solve the convex QP problem for the best-fit protein vector (**p**′****).

We defined a “relaxation” proteome budget (*P*_R_) variable to represent a pool of unspecified protein, accounting for uncertainty in both the proteomic data and the modeling workflow. Using the best-fit protein vectors, context-specific models were created for each sample by replacing Equations (4) and (18) with the following:


$$\begin{array}{c} P_{\text{total}}=P_{\text{HB}}+P_{\text{LA}}+P_{\text{R}} \end{array}$$ (23)

$$\begin{array}{c} P_{\text{R},\text{ub}}=s\underset{j}{\sum }p_{j}^{'}w_{j} \end{array}$$ (24)

$$\begin{array}{c} 0\leq P_{\text{R}}\leq P_{\text{R},\text{ub}} \end{array}$$ (25)

$$\begin{array}{c} p_{j}= \{\begin{array}{cc} 0\leq p_{j}\leq s\left(P_{\text{R},\text{ub}}/w_{j}\right), & p_{j}^{\text{data}}=\text{NaN}\\ \left(1-s\right)p_{j}^{'}\leq p_{j}\leq p_{j}^{'}+s\left(P_{\text{R},\text{ub}}/w_{j}\right), & p_{j}^{\text{data}}\geq 0 \end{array} \end{array}$$ (26)

with slack term (*s*) used to relax individual protein constraints, and to set the upper limit for the relaxation budget (*P*_R,ub_). This formulation deviates slightly from the OVERLAY pipeline [259], as the relaxation budget represented the mass equivalent of the total protein returned to the unspecified protein pool caused by relaxing lower bounds of best-fit proteome, and upper bounds were relaxed by using the molar equivalent of a portion of *P*_R,ub_, again determined by the slack term. Equation (18) was replaced with Equation (23) to prevent total protein content from exceeding 100% with the inclusion of the variable relaxation budget. Equation (4) was replaced with Equation (26) to reformulate the base proteome-constrained RBC-GEM reconstruction as context-specific models based on the samples.

Because the protein content of RBCs is primarily hemoglobin, strategies to obtain deep coverage of the low abundance RBC proteome often requires first depleting the hemoglobin [27, 28]. Furthermore, absence of proteomic evidence for a known RBC protein in the low-abundance proteome may be caused by technical and methodological reasons, as strongly indicated by the variability in the RBC proteome across studies (S1 Fig). We therefore assume that unmeasured RBC proteins likely escaped detection due to a very low protein abundance. Thus, the alterations to constraints through Equations (23–26) were necessary to prevent the uncertainty in much larger hemoglobin measurements from having an outsized influence on predicted protein allocation within the low-abundance proteome.

The slack term was determined via optimization of context-specific models representing mean and median protein abundances at each time point (Day 10, 23, and 42). Models were optimized at increasing slack values using the dual objective of maximizing flux through desired reactions (e.g., the Na^+^/K^+^-ATPase) while minimizing the utilized relaxation budget. Through Equations (23–26). The slack value was set as 0.03, a value determined to ensure solution feasibility by alleviating restrictions caused by unmeasured, yet essential, proteins for all context-specific models without significantly affecting results (S2 Fig).

Using the best-fit proteome vector for all 1848 samples of the RBC proteomic data, as well as for six additional representing mean and median protein abundances for each time point, we formulated 1854 context-specific proteome-constrained models for subsequent analyses. We computed the corresponding fluxome using proteome-constrained FVA [259] with the Na^+^/K^+^-ATPase set as objective reaction, and constrained to 0%, 50%, 90% and 99% of its maximum value. For each context-specific model, the effective minimum and maximum fluxes were computed for each reaction. For each reaction, the minimum and maximum total abundance of associated catalytic complexes was also computed, determined by optimizing the sum of abundances for corresponding forward and reverse “enzyme” entities. Spearman rank correlation coefficients (*ρ*) were computed between maximum flux and ‘enzyme’ abundance for all reactions, which were subsequently classified as abundance-dependent (0.8 ≥ *ρ*), abundance-correlated (0.5 ≤ *ρ* < 0.8), or abundance-independent (*ρ* < 0.5). Reaction without any gene associations (e.g., spontaneous), and blocked reactions, regardless of gene associations, were classified as always abundance-independent. Within each classification, both reactions and associated their proteins were grouped by metabolic category, with proteins further grouped if they acted on identical reaction sets.

All simulations performed were implemented in Python 3.11 using the COBRApy python package (version 0.29.0 [285,286]) and its implementation of algorithms for FBA/FVA [298–300].

## Supporting information

S1 Fig Comparison of proteomic evidence collected across 29 studies of the RBC proteome.
The proteomic evidence for proteins identified across all 29 studies of the RBC is visualized as a binary heatmap representing the detection (bright red) or absence (dark red) of proteins across individual studies.
(TIF)

S2 Fig Determination of slack value for relaxation of the proteome budget.
Models that represent the mean and median protein abundance values were simulated at different slack values, maximizing flux through the sodium-potassium pump while minimizing the relaxation budget utilized.
(TIF)

S1 File The RBC-GEM model in Systems Biology Markup Language.
The fully annotated RBC-GEM reconstruction is provided in Systems Biology Markup Language (SBML). The RBC-GEM reconstruction is also found in the BioModels repository (ID: MODEL2410170001).
(XML)

S2 File Supplementary tables and data for the RBC-GEM reconstruction.
A table of contents containing the table titles and legends is included on the first sheet.
(XLSX)

S3 File The MEMOTE score for RBC-GEM 1.2.0.
The RBC-GEM 1.2.0 reconstruction passes the major MEMOTE tests for quality assurance and control tests with a score of approximately 83%.
(TIF)

S4 File The RBC-GEM metabolic network map in multiple file formats.
The map is provided in multiple different formats for accessibility, including a browsable HTML file, a PDF file, and the original JSON file for Escher. The EscherConverter 1.2 software was used to generate analogous SBML layout and SBGN files from the map JSON.
(ZIP)

S5 File Supplementary tables and data for the RBC-GEM connectivity calculations.
A table of contents containing the table titles and legends is included on the first sheet.
(XLSX)

S6 File Supplementary tables and data for proteome-constrained modeling of RBCs.
A table of contents containing the table titles and legends is included on the first sheet.
(XLSX)

S7 File Representative REDS RBC Omics proteome-constrained RBC-GEM model in Systems Biology Markup Language.
The fully annotated proteome-constrained RBC-GEM genome-scale model is provided in Systems Biology Markup Language. Prefixes on identifiers are used to denote the additional model objects applied to formulate the proteomic constraints. The proteome-constrained RBC-GEM reconstruction is also found in the BioModels repository (ID: MODEL2501130001).
(XML)
