# Supplementary figures and images for: RBC-GEM: A genome-scale metabolic model for systems biology of the human red blood cell
Source: PLoS Comput Biol. 2025 Mar 12;21(3):e1012109. doi: 10.1371/journal.pcbi.1012109 (PMC11925312; doi:10.1371/journal.pcbi.1012109)

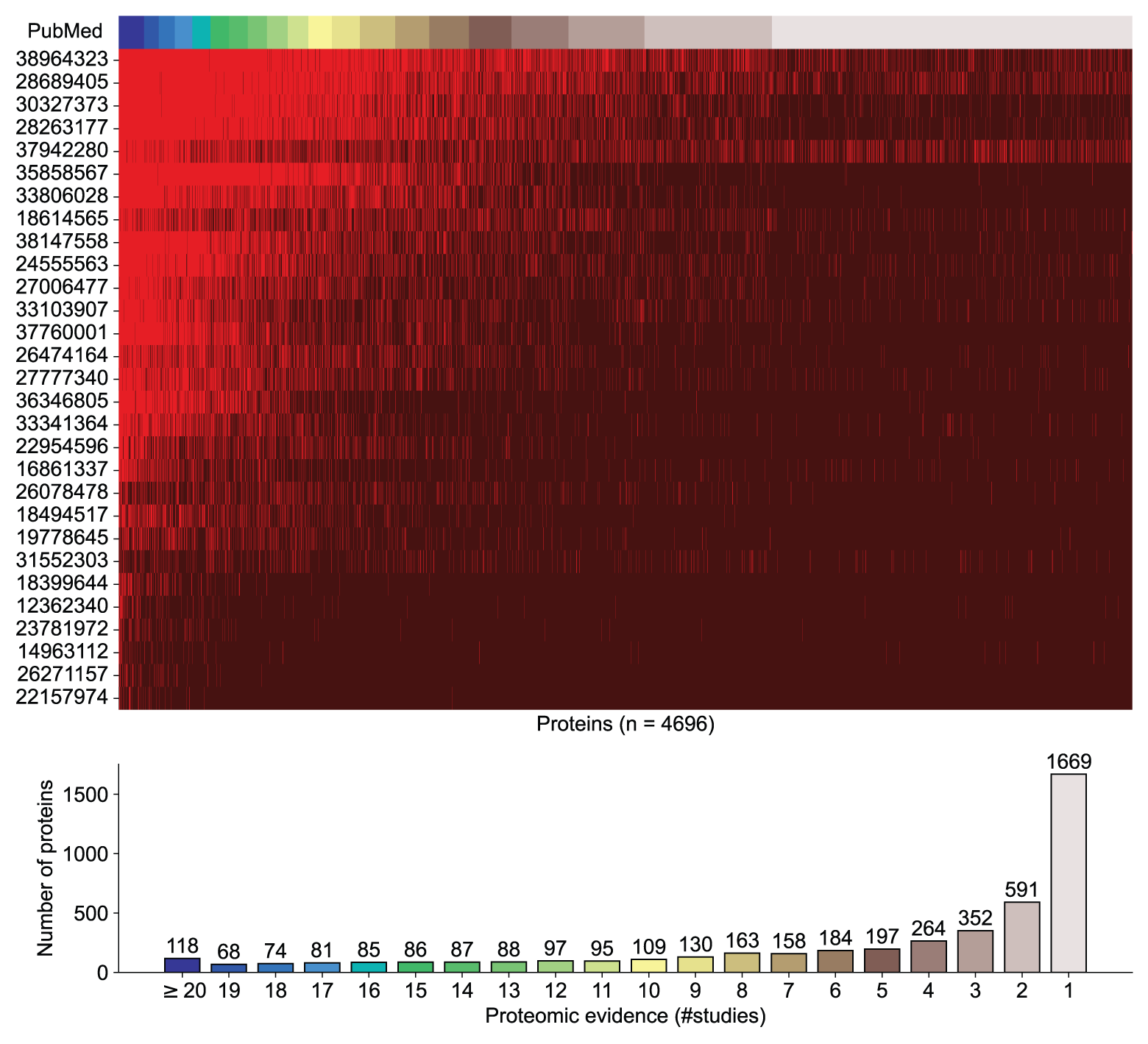

Supplement: S1 Fig — The proteomic evidence for proteins identified across all 29 studies of the RBC is visualized as a binary heatmap representing the detection (bright red) or absence (dark red) of proteins across individual studies. (TIF) [file pcbi.1012109.s001.tif]

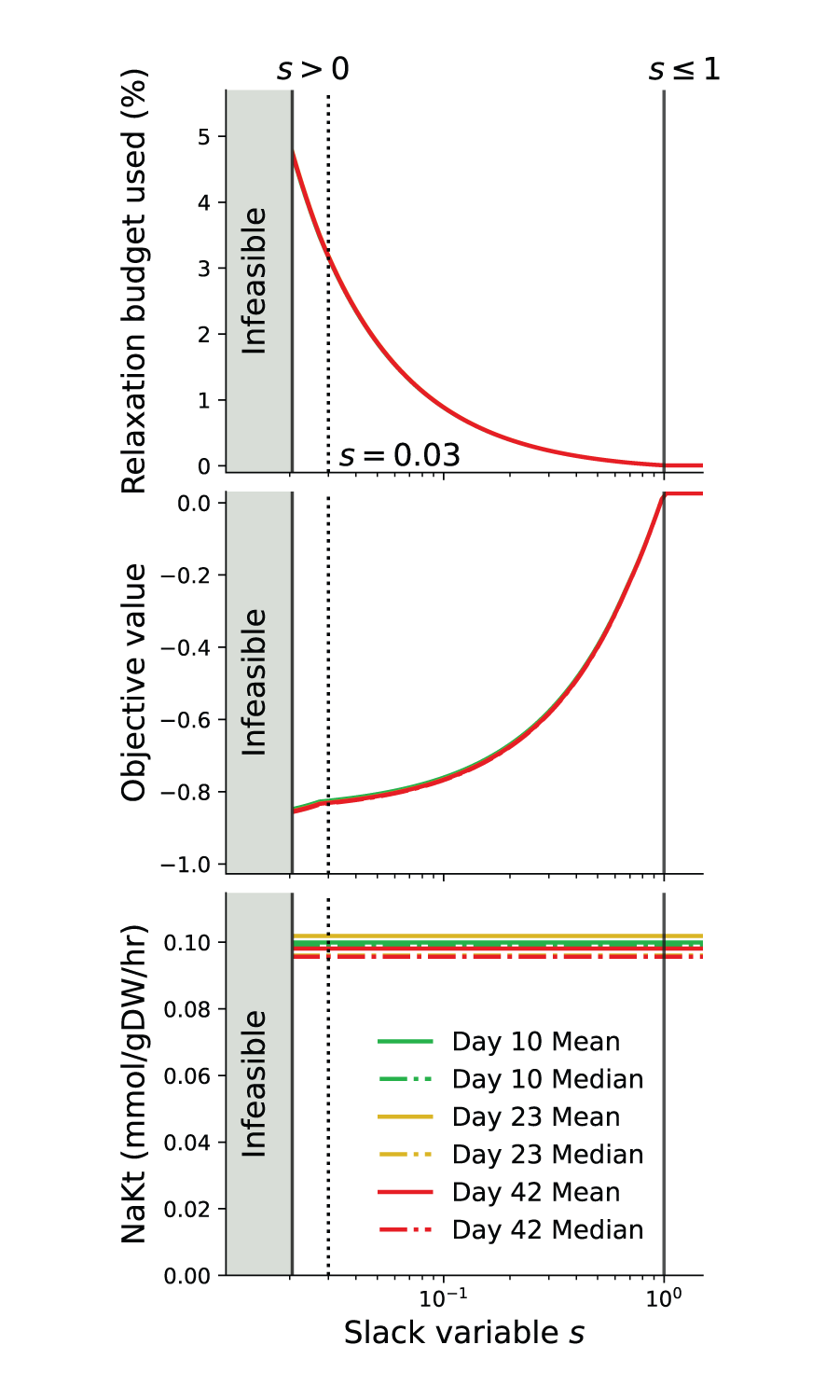

Supplement: S2 Fig — Models that represent the mean and median protein abundance values were simulated at different slack values, maximizing flux through the sodium-potassium pump while minimizing the relaxation budget utilized. (TIF) [file pcbi.1012109.s002.tif]

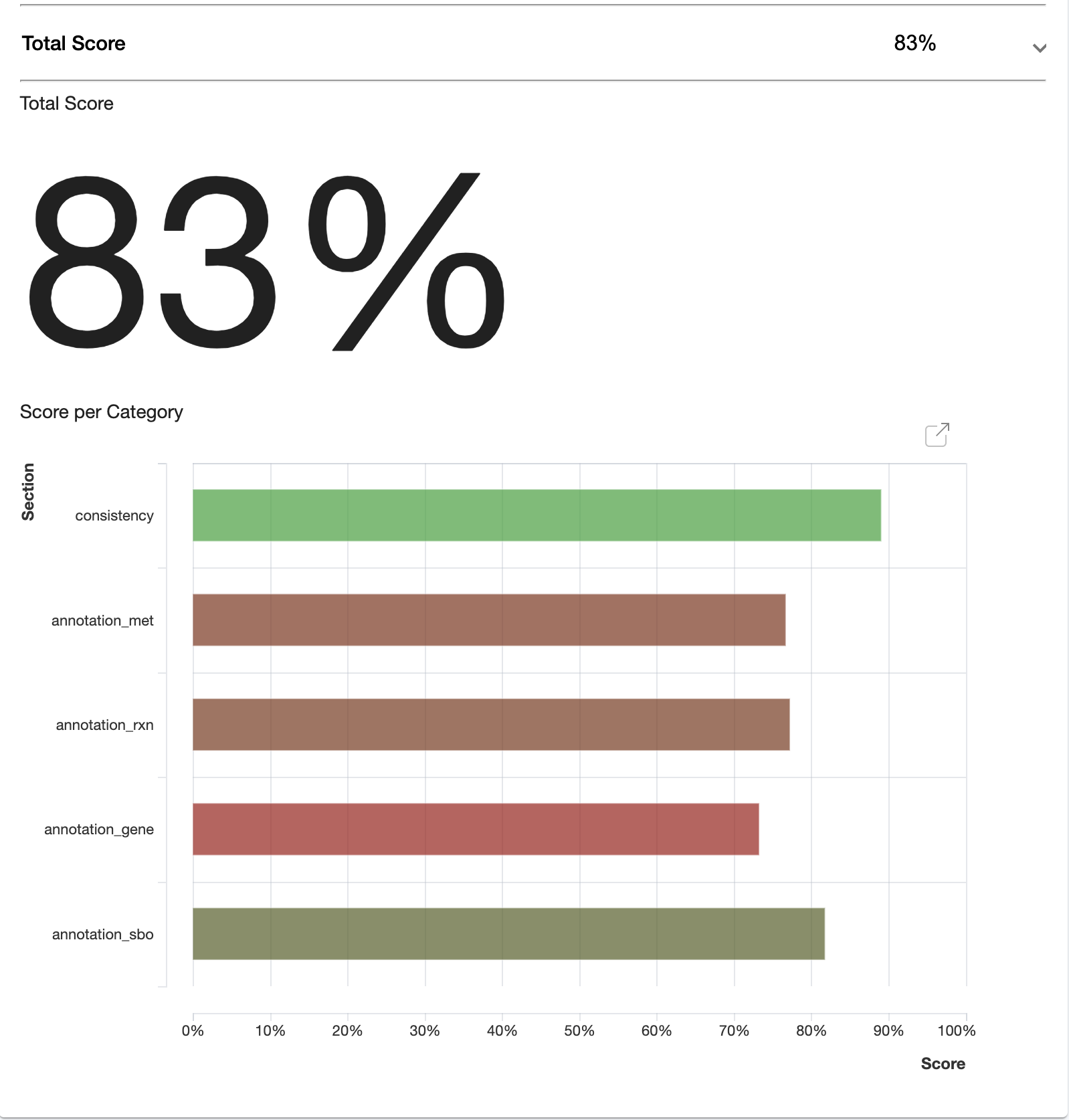

Supplement: S3 File — The RBC-GEM 1.2.0 reconstruction passes the major MEMOTE tests for quality assurance and control tests with a score of approximately 83%. (TIF) [file pcbi.1012109.s005.tif]

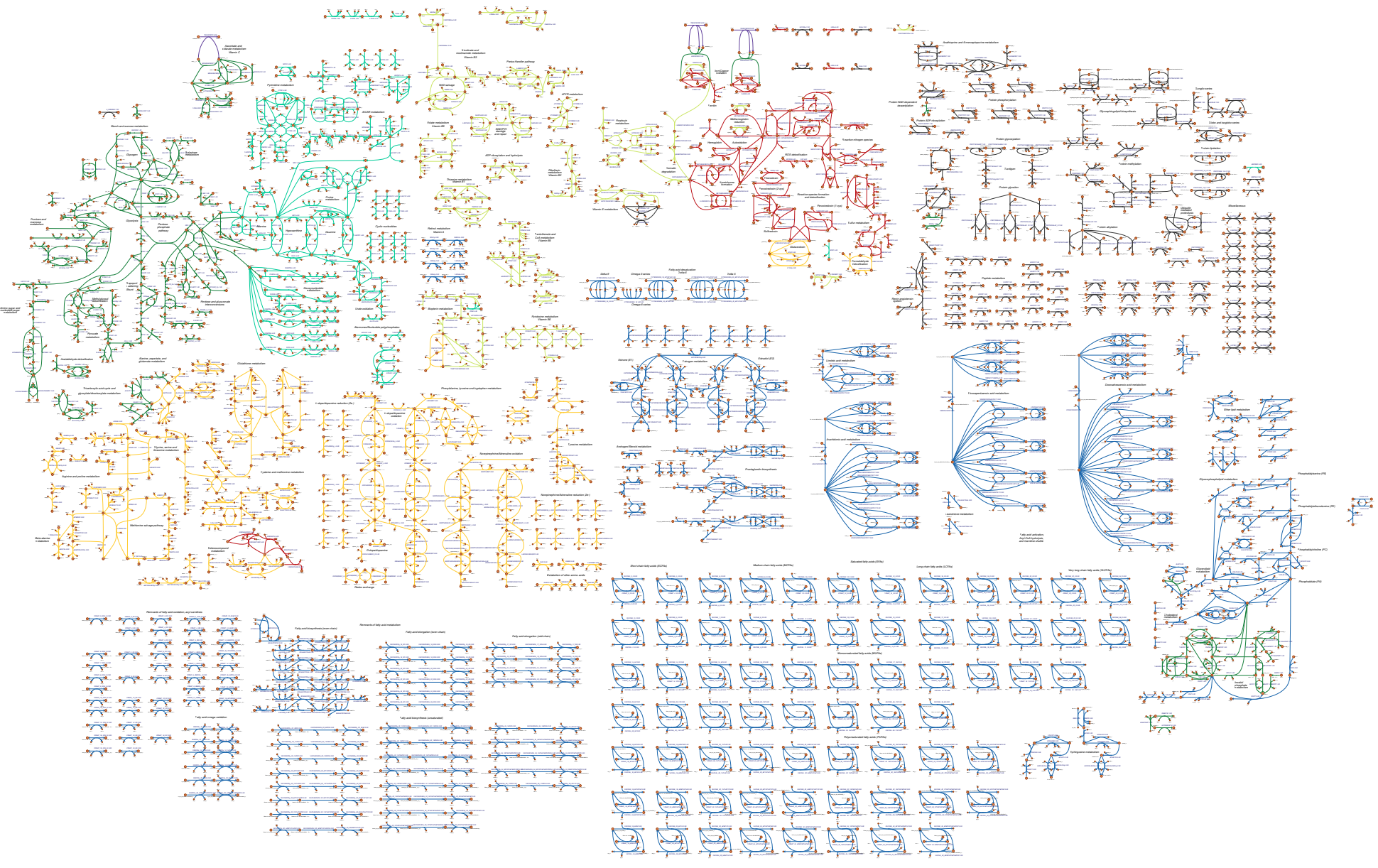

Supplement: S4 File — The map is provided in multiple different formats for accessibility, including a browsable HTML file, a PDF file, and the original JSON file for Escher. The EscherConverter 1.2 software was used to generate analogous SBML layout and SBGN files from the map JSON. (ZIP) [file pcbi.1012109.s006.zip › S4_File/RBC-GEM.full.map.pdf]
